# The role of creatine kinase in distinguishing generalized tonic–clonic seizures from psychogenic non-epileptic seizures (PNES) and syncope: a retrospective study and meta-analysis of 1300 patients

**DOI:** 10.1186/s42466-023-00286-0

**Published:** 2023-10-12

**Authors:** Ramy Abdelnaby, Anas Elgenidy, Jan Heckelmann, Mahmoud Mostafa Bedewy, Ahmed Samy Shabib, Mohamed Ayman Ebrahim, Khaled Abdelmoneim Elmenawi, Imene Maallem, Merna Wagih Youssef, Abdelrahman M. Attia, Mostafa Hossam Moawad, Khaled Ashraf Mohamed, Ahmed Gaballa

**Affiliations:** 1 Department of Neurology, RWTH Aachen University, Aachen, Germany; 2 Faculty of Medicine, Cairo University, Cairo, Egypt; 3 Faculty of Medicine, Mansoura University, Mansoura, Egypt; 4 Faculté de Pharmacie, 23 Avenue Maquis du Grésivaudan, 38700 La Tronche, Grenoble, France; 5 Faculty of Medicine, Alexandria University, Alexandria, Egypt; 6 Faculty of Pharmacy, Clinical Department, Alexandria University, Alexandria, Egypt; 7 Faculty of Medicine, Suez Canal University, Ismailia, Egypt; 8 Department of Epileptology (Krankenhaus Mara), Medical School, Bielefeld University, Campus Bielefeld- Bethel, Bielefeld, Germany

**Keywords:** Epileptic seizures, Psychogenic non-epileptic seizures, Syncope, Generalized tonic-colonic seizure, Creatine kinase

## Abstract

**Background/aim:**

As the clinical differentiation between epileptic seizures, psychogenic non-epileptic seizures (PNES), and syncope depends mainly on a detailed report of the event, which may not be available, an objective assessment of a potential biochemical analysis is needed. We aimed to investigate whether serum creatine kinase (CK) could be used to differentiate epileptic seizure from PNES and syncope and to assess the strength of evidence present.

**Methods:**

We directed a retrospective cohort study coupled with a systematic review and meta-analysis of studies that measured CK in patients with epilepsy, PNES, syncope, and healthy controls.

**Results:**

The cohort study, which traced 202 patients, showed that the CK level was significantly higher 48 h after the event in the epilepsy group versus patients with syncope (*p* < 0.01) Along with 1086 patients obtained through a database search for meta-analysis, CK level compared to different types of seizures from PNES was higher in epileptic seizure patients with a mean difference of 568.966 mIU/ml (95% CI 166.864, 971.067). The subgroup analysis of CK showed that it was higher in GTCS compared to syncope with a mean difference of 125.39 mIU/ml (95% CI 45.25, 205.52).

**Discussion:**

Increased serum levels of CK have been associated mainly with epileptic seizures in relation to non-epileptic events. However, further studies would try to explore the variation in measurements and any other potential diagnostic marker.

**Conclusion:**

The cohort study shows that the CK level in epilepsy seizures is higher after 48 h from the event compared to syncope. Moreover, the meta-analysis results show the present diagnostic utility of CK and its importance to be used in accordance with a detailed report of the event.

**Supplementary Information:**

The online version contains supplementary material available at 10.1186/s42466-023-00286-0.

## Introduction

The differential diagnosis of transient loss of consciousness requires a differentiation among epileptic seizures, psychogenic non-epileptic seizures (PNES), and syncope. As all diseases have different pathophysiology and therapeutic approaches, precise classification is essential for subsequent management.

Syncope represents a sudden loss of consciousness, linked to the inability to maintain postural tone, with instant recovery but exposed to complications of falls. Among the causes of syncope, the facilitated neural reflex known as vasovagal syncope is the most common. Other causes include cardiac origin, orthostatic hypotension, carotid sinus hypersensitivity, neurological, and endocrinological causes [1]. Patients with PNES, presenting with episodes of movement, sensation, or behaviors that resemble epilepsy, are at risk of iatrogenic complications through antiseizure medications' side effects and unnecessary hospitalizations, especially in intensive care units with potential complications because of invasive medical procedures [2]. Therefore, early identification would help prevent such complications. While the diagnostic work-up of transient loss of consciousness is mainly based on a detailed report of the event, difficulties may arise in distinguishing between different causes, particularly when a detailed report of the event is unavailable. Additionally, there is a possibility of co-occurrence of both disorders, further complicating the diagnostic process [2].

As PNES is suspected clinically, and the differentiation against ES is mainly through video electroencephalography (VEM) recording [3], we included studies which included either detailed reports of the event or were identified through VEM. However, the later method is time-consuming and requires a PNES to occur while the recording [4]. Although postictal serum creatine kinase (CK) in clinical settings has varying degrees of fluctuations, its use in the differentiation between tonic–clonic epileptic seizures from PNES and syncope could be time saving and represent a dependable clue in the process of differentiation [5]. Therefore, we conducted this systematic review and meta-analysis to investigate the use of this marker which is easily obtained through blood samples.

## Methods

### Cohort study

We conducted a retrospective cohort study on patients who had epilepsy, only generalized tonic-colonic seizure (GTCS), or syncope retrieved from the electronic database of the Neurology department, RWTH University Hospital of Aachen, Germany. Ethical approval was obtained from the Ethical Committee of the Faculty of Medicine of RWTH University Hospital of Aachen (EK 031-22), and because of the retrospective nature, the ethics committees waived the need for patient-signed consent. This report follows the Strengthening the Reporting of Observational Studies in Epidemiology (STROBE) guidelines [6].

The authors collected the following patient data: age, sex, date of presentation, and CK level from 24 and 48 h after the event. For continuous variables, data were assessed for normality of distribution visually first by histogram and then confirmed by Shapiro-Wilks Normality Test (where *p* value > 0.05 indicates non-normality). Continuous variables were represented in the form of Median (IQR). For categorical variables, data were presented as the number of patients and percentage (%). The comparison of continuous variables was conducted using an independent t-test, while the Chi-square test was used to compare categorical variables between the two groups. Area under the Curve (AUC) and F1 score were used to evaluate the accuracy of serum creatine kinase (CK) as a diagnostic marker. A *p* value of ≤ 0.05 was considered statistically significant. Statistical analysis was conducted using R programming language version 3.6.3. and SPSS version 25 (IBM Corp. Released 2017. IBM SPSS Statistics for Windows, Version 25.0. Armonk, NY: IBM Corp). *p* value < 0.05 is considered.

### Review study

#### Data sources and searches

**Fig. 1:**
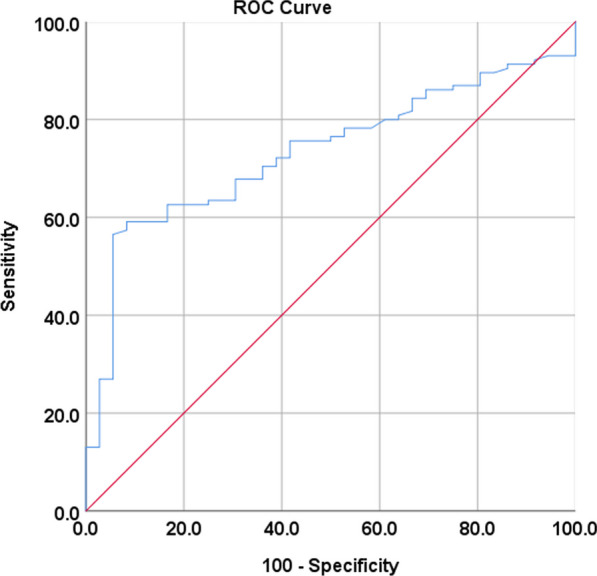
Receiver operator characteristic (ROC) curve for CK level in day 2 GTCS versus Syncope cases

For the aim of identifying relevant studies, we conducted an online literature systematic search on the following databases: MEDLINE (through PubMed), Scopus, Web of Science, and Embase up until April 1st, 2022 with no restrictions on language, date of publication or study design using the "title and abstract" domain to reach all the studies related to the measurement of serum CK levels in epileptic patients and non-epileptic patients including PNES, syncope or healthy control group. The synonyms of our search strategy were retrieved from the Medical Subject Headings terms (MeSH terms) for seizure and creatine kinase terms and were combined by “OR” and “AND” Boolean operators according to the recommended method described in Cochrane Handbook for Systematic Reviews of Interventions (Chapter 4.4.4) [7] as detailed in (Additional file 1: Table S1). Our study was conducted according to the Preferred Reporting Items for Systematic Reviews and Meta-Analyses (PRISMA) guidelines, 2020 version [8]. The details of the screening process, included and excluded studies, are shown in the PRISMA flow diagram (Fig. 1).

#### Eligibility criteria

We included studies that met the following criteria: full-text articles published in international peer-reviewed journals measuring creatine kinase (CK) serum levels in patients who suffered from different kinds of epilepsy, non-epileptic events such as PNES, syncope, or healthy control subjects and provided numerical data of CK measurements for each group separately; relevant articles regardless of the original language of publications. Studies that didn't provide the needed numerical data were not included in the quantitative synthesis but were included in the qualitative synthesis. Our exclusion criteria were case reports, letters to the editor, and animal studies. Duplicate records were removed using EndNote (version 8.2) [9].

#### Selection and screening

Five Authors (I.M., M.H., M.A.E., M.W.Y., and A.S.M.) screened studies by title and abstract according to our inclusion and exclusion criteria to identify eligible articles, then they reviewed articles that were selected for full-text screening to determine the final list of eligible studies. Studies that fulfilled the eligibility criteria were included in the qualitative and quantitative analyses. Any disagreements between the authors on whether to include or exclude a certain study were resolved through consultation and discussion with a senior author (R.A.). Reference lists of identified relevant articles were cross-referenced for other articles of interest and were retrieved for full-text screening to assess their eligibility and to ensure literature saturation.

#### Quality assessment

Three authors (M.H., M.A.E., and A.S.M.) carried out the process of quality assessment of the included studies blindly and independently. Some differences were encountered, and they were referred to a third author (R.A.) who also took the decision in a blinded independent fashion. Three types of studies were included in our systematic review: case–control, cross-sectional, and cohort studies. For case–control studies, we used the Newcastle–Ottawa Quality Assessment Scale (NOS) for case–control studies [10]. NOS for case–control studies is a star-based scale that evaluates each study in three main categories: selection of study population, exposure, and comparability. A scale of a maximum number of stars equal to 9 is used. Studies with a score of 7–9 stars were considered high-quality studies, studies with a score of 4–6 stars were considered moderate-quality, and studies with a score of 0–3 stars were considered low-quality.

For cross-sectional and cohort studies, we used the National Institute of Health (NIH) quality assessment tool for observational cohort and cross-sectional studies [11]. This tool is made of 14 yes/no questions in addition to not applicable not reported information sufficient for answering a specific question. Studies with a score of ≥ 9 or more were considered good quality, while those with a score of 5–8 were considered fair quality, and those with a score of less than 5 were considered poor quality.

#### Data extraction

Data from included studies were extracted independently by five authors (M.A.E., I.M., K.A.E., M.W.Y., M.B.) in a pre-defined Excel sheet where the following information was extracted: Study-related variables (authors, year of publication, study country and design, time of CK measurement); Patient-related variables (age, sex); epilepsy type outcome variables (basal CK serum levels values and type of epileptic attack); and type of non-epileptic attack (PNES, type of syncope and controls). If these data were not available or the methods required clarification, the corresponding authors of included studies were contacted via e-mail requesting additional unpublished data. Articles were excluded from further analysis when corresponding authors could not be contacted or could not provide the information on request. Studies published in a language other than English were translated.

#### Data analysis and synthesis

This analysis pooled the mean difference between epileptic and non-epileptic patients (PNES, syncope, and healthy controls), comparing the rates of CK serum levels. We performed two types of analyses: a double-arm meta-analysis and a single-arm meta-analysis using OpenMetaAnalyst [12] software to compare the mean serum CK levels values in epileptic patients and non-epileptic patients by calculating the pooled mean difference in serum CK level values. Also, we conducted a subgroup analysis by stratifying the studies according to CK serum levels measured and examined the difference in CK serum levels between epileptic patients and non-epileptic types. Values reported as median and interquartile range or range were converted to mean and standard deviation using the method proposed by Hozo et al. [13]. The random effects model of the DerSimonian and Laird [14] method was implemented to account for heterogeneity. Heterogeneity was assessed by the Chi-square test and the *I*^2^ statistic, with *p* < 0.05 proving significant heterogeneity and *I*^2^ > 50% indicating substantial heterogeneity. Sensitivity analysis was carried out to examine the effect of elimination of each study on the overall results. Moreover, a leave-one-out meta-analysis was performed to evaluate the effect of a single study on the pooled results. Publication bias assessment was not possible due to the small number of included studies in each meta-analysis [7].

## Results

### Cohort study

Two hundred and two patients' data were retrieved from the database, the distribution of the different patients in the three groups in addition to Age and sex distribution was summarized in Table 1. About 74.7% of the patients had GTCS, and 25.3% had Syncope, the median age distribution was 58, and 74 for GTCS, and Syncope respectively.

**Table 1:** Age, sex distribution, and CK levels in different groups

	Epilepsy (n = 151)	Syncope (n = 51)	*p* value
Age in years (median [IQR])	58.00 [38.00, 75.00]	74.00 [53.00, 84.00]	0.003**
Sex (%)			
Male	83 (55.0)	26 (51.0)	0.621
Female	68 (45.0)	25 (49.0)	
CK level after 24 h (median [IQR])	114.00 [67.50, 195.00]	91.00 [65.50, 130.00]	0.061
CK level after 48 h (median [IQR])	213.00 [86.00, 509.50]	79.00 [48.50, 128.50]	< 0.001***

There was no significant difference in sex distribution between the two groups (*p* = 0.621). Statistically significant age distributions were noted between GTCS and syncope patients with a (*p* = 0.003) (Table 1).

CK level in each group was measured and reported after 24 and 48 h and represented in the form of Median (IQR), A comparison of CK levels between GTCS and syncope was conducted all through the 48 h as shown in Table 1. In addition, we reported the results of each group compared to 2 different age groups of more or less than 40, as mentioned in Table 2. There was no statistically significant difference between both age groups (above or below 40) in either GTCS or syncope.

**Table 2:** CK level in Epilepsy and syncope (above 40 years vs below)

Day	Above 40	Below 40	*p* value
Day 1	106 (20–1240) (N = 110)	123 (22–790) (N = 40)	0.75
Day 2	216 (8–8851) (N = 86)	196 (24 -1796) (N = 29)	0.45
Day 1	87 (25–664) (N = 36)	111 (42–197) (N = 15)	0.21
Day 2	74 (35–936) (N = 26)	81 (34–197) (N = 10)	0.48

There was no statistically significant difference in CK level between GTCS and syncope patients after 24 h (*p* = 0.061). CK level was statistically significant higher after 48 h in the GTCS versus the syncope (*p* < 0.001).

The area under the curve (AUC) for CK level on day 2 for diagnosis of GTCS versus Syncope was 0.73 shown in Fig. 1 with 56.5% Sensitivity and 94.4% Specificity with positive predictive value (PPV) = 56.5% and negative predictive value (NPV) = 94.4%, meaning that the accuracy of CK level to diagnose GTCS versus Syncope was fair confirmed by measuring F1 score of 0.58, which is commonly seen as an OK value (Fig. 1).

### Secondary results:

**Fig. 2:**
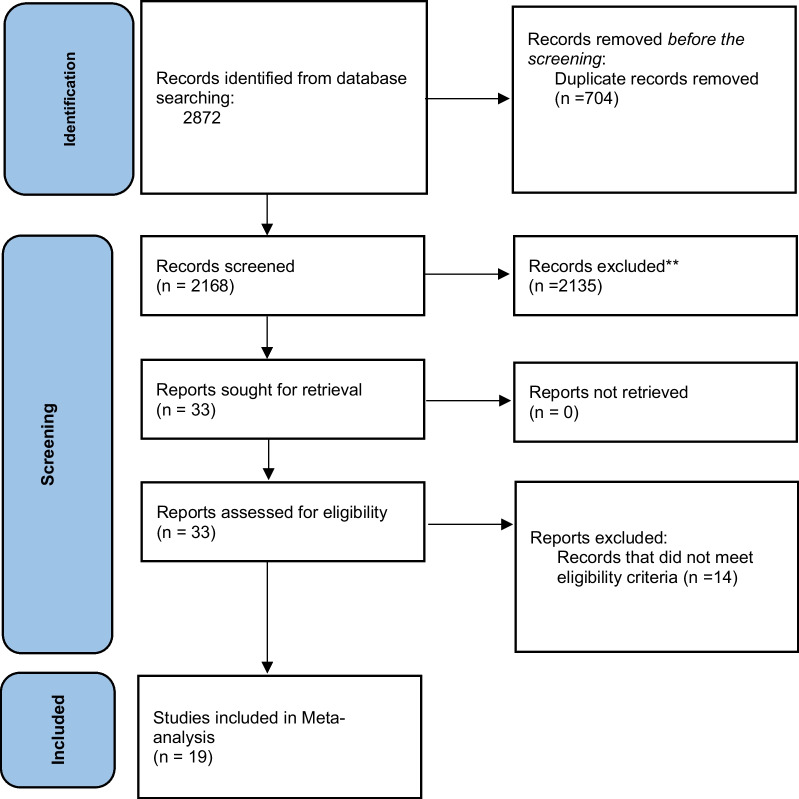
PRISMA flow diagram of the included studies and screening process

According to the search strategy we followed, 2872 studies were found. Among the 2168 screened ones, 2149 were excluded from further analysis leaving 19 -in addition to our primary study- studies which were included in this systematic review and meta-analysis. The details of the screening process included and excluded studies are shown in the PRISMA flow diagram (Fig. 2).

### Characteristics of the included studies

Of the 16 studies included in the meta-analysis (quantitative analysis), 1086 patients were enrolled in our study; of which 733 patients suffered from epileptic seizures of different types, 71 patients had PNES, 195 patients suffered from syncope and 87 were healthy control patients as shown in (Additional file 1: Table S2 and S3). Also, three studies were included in the systematic review (qualitative analysis) and were presented in (Additional file 1: Table S4).

### Quality assessment

Using the National Institute of Health (NIH) quality assessment tool for observational cohort and cross-sectional studies, we found four good-quality studies and seven fair-quality studies, as shown in (Additional file 1: Tables S5 and S6) case–control studies using Newcastle–Ottawa quality Scale (NOS), we found three high-quality studies, three moderate quality studies, and two low-quality studies mainly due to the representativeness of the cases and selection of controls domains as shown in (Additional file 1: Table S7).

### Epilepsy versus PNES

#### GTCS versus PNES

Three studies were enrolled in this analysis [15–17] to compare CK levels in GTCS versus PNES patients with a total of 237 patients for the GTCS and 59 patients for PNES. CK level was higher in GTCS patients with a mean of 923.064 mIU/ml (95% CI 65.578, 1780.549); *p* value was significant (*p* < 0.01) with substantial heterogeneity (*I*^2^ = 98.62%) as shown in Additional file 2: Fig. S1).

#### Different types of seizures versus PNES

Four studies [15–18] with a total of 317 patients for different types of epileptic seizures and 71 patients for the PNES group. CK level was higher in epileptic seizure patients with a mean of 568.966 mIU/ml (95% C.I 166.864, 971.067) with a *p* value of < 0.01. Significant heterogenicity was found (*I*^2^ = 97.88%). Results are shown in (Additional file 2: Fig. S2). To address the significant heterogeneity, a sub-group analysis was conducted for the same studies according to age, as shown in (Additional file 2: Fig. S2 and Additional file 1: Table S8).

### Epilepsy versus healthy controls

#### GTCS versus healthy controls

Three studies [15, 17, 19] with a total of 90 patients for the GTCS and 71 patients for the control. CK level was higher in GTCS with a mean of 670.89 mIU/ml (95% CI − 198.66, 1540.44); *p* value was insignificant (*p* = 0.13), and significant heterogenicity was detected (*I*^2^ = 99.54%). Results are shown in (Additional file 2: Fig. S3). To investigate sources of heterogeneity, a sub-group analysis of the same studies according to the country was performed as shown in (Additional file 2: Fig. S3 and Additional file 1: Table S9).

#### Different types of seizures versus healthy controls

Four studies [15, 17–19] with a total of 142 patients for different types of epileptic seizures and 87 patients for the control group. CK level was higher in epileptic seizures with a mean of 515.19 mIU/ml (95% C.I 86.49, 943.90); *p* value was significant (*p* < 0.01), and significant heterogeneity was detected (*I*^2^ = 99.55%). Results are shown in (Additional file 2: Fig. S4). Sub-group analysis for the same studies according to the study country was performed, as shown in (Additional file 2: Fig. S4 and Additional file 1: Table S10).

### GTCS versus syncope

Six studies (Current study 2022, [17, 20–23] were included in the analysis. The studies were sub-grouped according to the time of measurement, with a total number of 439 measurements for the GTCS and 248 measurements for the syncope which showed that CK was higher in GTCS compared to syncope with a mean difference of 125.39 mIU/ml (95% CI 45.25, 205.52) (Additional file 2: Fig. S5). To investigate sources of heterogeneity, a subgroup analysis by study country with a total number of 600 measurements for the GTCS and 270 measurements for the syncope with a mean difference of 164.18 (95% CI 55.73, 272.64) (Additional file 2: Fig. S5), and according to age with a total number of 600 measurements for GTCS and 270 measurements for syncope with a mean difference of 164.18 (95% CI 55.73, 272.64) (Additional file 2: Fig. S5). The results of these subgroup analyses are also presented in (Additional file 1: Table S11).

### Mean CK level in GTCS patients

According to the type of measurement, mean CK level was higher at 0–6 h (721.6 mIU/mL) followed by day 2 post-ictal (249.7 mIU/mL), then 24 h postictal (207.55 mIU/mL) as shown in Additional file 2: Fig. S6 and Additional file 1: Table S12. Regarding the measurements of CK on the days after the event, it was found to be highest on day 6 (744.49 mIU/mL), followed by day 5 (738.55 mIU/mL), day 3 (577.8 mIU/mL), day 4 (359.65 mIU/mL) and finally day 7 (156.48 mIU/mL) as shown in Additional file 2: Fig. S6 and Additional file 1: Table S13.

Subgroup analysis of mean CK level measured at 0–6 h following seizures showed that it was higher in patients below 40 years old compared with older than 40 years old (721.6 vs 570.17 mIU/mL), highest in studies conducted in Switzerland (1488.5 mIU/mL) and lowest in studies conducted in Israel (116 mIU/mL), as well as it was highest in studies measuring CK by random kit (2577 mIU/mL) and lowest in studies using automatic analyzer (146.5 mIU/mL) (Additional file 2: Fig. S7 & Additional file 1: Table S14).

Regarding mean CK level measured at 1 day postictal, it was higher in patients above 40 years compared to those below 40 years (334.8 vs 109.03 mIU/mL), and it was higher in the USA compared to Germany (1324.92 vs 201.93 mIU/mL).

The subgroup analysis on the second day postictal showed that mean CK level was higher in patients older than 40 years compared to those below 40 years, (350.13 vs 196.48 mIU/mL), and higher in the USA compared to Germany and Israel (472.55 vs 214.59, and 217 mIU/mL, respectively) (Additional file 2: Fig. S8 and S9 respectively & Additional file 1: Tables S15 and S16).

### Sensitivity analysis

#### GTCS versus PNES

The omission of a study [17] seems to have a major influence on the estimation of the overall effect size (mean = 1308.49; CI 1159.32–1457.66) as well on the heterogeneity (*I*^2^ = 0%; *p* = 0.79) as shown in (Additional file 2: Fig. S1). While other sensitivity analyses didn’t seem to have any significant effect on overall results or heterogeneity.

#### GTCS versus healthy control

As Belton 1967 et al. [19] is the only included study that measured serum CK levels in children, we conducted a sensitivity analysis by removing it from the analysis, we noticed an insignificant rise in mean CK level (mean = 983.47; CI − 572.74 to 2539.69) with nearly no change in the previously observed heterogeneity (*I*^2^ = 99.7%; *p* < 0.01) as shown in (Additional file 2: Fig. S3).

#### Different types of epileptic seizures versus healthy control

We conducted a sensitivity analysis by omitting Belton 1967 et al. [19], for the same reason mentioned previously. Serum CK levels between the two groups turned out to be insignificant with substantial heterogeneity after omitting Belton 1967 et al. (mean = 679.68; CI − 221.06 to 1580.42; *I*^2^ = 99.63) as shown in (Additional file 2: Fig. S4). Another sensitivity analysis was performed by eliminating Ijaz 2020 et al. [15], and we showed a drop in mean serum CK level and heterogeneity, as well (mean = 82.82; CI 18.86 to 146.79; *I*^2^ = 78.05%) as shown in (Additional file 2: Fig. S4).

### Leave-one-out analysis

#### Leave-one-out analysis for the mean CK level in the first 6 h for GTCS patients.

A significant change in the overall mean was noticed when Ijaz 2020 was removed [10], as shown in (Additional file 2: Fig. S10).

#### Other leave-one-out analyses

We performed leave-one-out analyses for the poor-quality studies, which were addressed by quality assessment tools, but no significant changes were noticed.

## Discussion

A diagnostic challenge can arise when differentiating between epileptic seizure, PNES, and syncope. The coexistence of both PNES and epileptic seizures can make it difficult to distinguish between these two disorders [2].

The diagnostic level of PNES varies with certainty. A possible diagnosis is based mainly on any witness or self-report. A probable diagnosis is established by a clinician who reviewed video recordings or a case typical of PNES. Subsequently, the diagnosis becomes clinically established by a seizure disorder (on video or in person), showing semiology typical of PNES, while not on EEG, requiring EEG testing to document the PNES. Therefore, the requirement for a postictal test to escalate the diagnostic certainty of PNES is highly valuable to distinguish between ES, PNES, and syncope. Although the evidence for the diagnostic use of postictal serum creatine kinase (CK) in clinical settings is inconsistent, it has been proposed as a potentially useful marker to discriminate between GTCS and PNES [5].

Creatine kinase is an essential part of muscle metabolism. It is typically present in high amounts in metabolically active cells, such as neurons and skeletal muscles [18]. CK was found to be elevated in both epileptic and PNES. This can be attributed to the intense muscle contractions that cause the release of CK into the blood in epileptic and PNES individuals due to some sort of muscular injury [15].

Neufeld et al. showed that a considerable spike increase in CK levels occurs after 48 h of seizure despite the initial normal value. They presented that an increase of at least 15 U/L is very predictive of an epileptic event [22]. Brigo et al. and Nass et al. showed a significant CK rise in 40% to 60% of GTCS in an emergency setting [5, 24]. On the other hand, the predictive diagnostic significance of CK elevations revealed substantial CK rises only after 14 to 19% of the GTCS [18].

The reason CK levels are greater in emergency room studies than in research using video-EEG monitoring is unknown. Nass et al. suggested that one explanation might be because emergency room studies included GTCS due to other reasons than idiopathic epilepsy, including drugs, for example, alcohol, as well as possible falls and injuries that weren't reported or seen. Instead of using a hospital bed, unprotected lying on hard surfaces might potentially be a factor as well [25]. In addition, throughout the studies, PNES is reached out through a workup that was meant initially to establish the diagnosis of seizure disorder through a detailed report of the event. Different levels of diagnostic certainty range from possible diagnosis by having a witness to documented PNES through which an experienced clinician observes a patient with a semiology of PNES while on EEG [26]. An inconsistent approach to diagnosing PNES would result in fluctuating numbers as the present gold standard method requires a present attack accompanied by an EEG. While CK is increased through vigorous muscle contraction, there is no etiological correlation among the PNES, ES, and syncope for the rise of CK. The reported number of patients with PNES patients was much lower compared to any other group, which may have led to misrepresentation of the limbs while conducting the analysis, denoting the substantial level of heterogeneity.

Other serum biomarkers were utilized in previous studies to differentiate between GTCS, PNES, syncope, and non-GTCS such as lactate. Lactate was found to be increased in GTCS patients compared to the other three causes of loss of consciousness (PNES, syncope, and non-GTCS). However, no significant difference was found between those three in lactate level, so it can be only used to differentiate between GTCS and other causes of loss of consciousness [27]. On the other hand, there are some limitations for lactate use in this differential diagnosis. A prolonged tourniquet application may deceitfully raise the serum lactate levels. Thus, it is advantageous to take blood samples from unconscious patients without using a tourniquet [28]. Moreover, before attributing high serum lactate to GTCS, it's crucial to consider other reasons for elevated lactate including sepsis, shock, trauma, hepatic dysfunction, and some drugs like metformin [29, 30].

On the other hand, other diagnoses should be ruled out when CK level is elevated including rhabdomyolysis that may result in acute kidney injury. However, seizures may be associated with rhabdomyolysis due to extreme muscle activity, falls or trauma associated with seizures. Ammonia may also be increased due to muscle breakdown because of the same reasons [31]. Rhabdomyolysis can be diagnosed when the CK level is five times the upper limit of normal readings [32] and renal injury is more likely to develop when CK > 5000 IU/L, however, this is not always the case without the presence of additional risk factors [33].

Compared to the latest systematic review by Brigo et al. [34] on the same topic, which provided only descriptive findings, our study included more primary studies in addition to the meta-analysis we performed which potentiates the level of evidence. Moreover, Bringo et al. did not include syncope to the included studies. On the other hand, we conducted a primary study to differentiate between syncope and GTCS based on the database from our institution and included syncope in our search strategy for the meta-analysis as well. Furthermore, their sample size was small, and they recommended further studies to be able to pool the results in a further meta-analysis.

Some studies stated that the level of CK elevation could be multifactorial. It is more likely to be caused by prolonged seizures, serial seizure, and status epilepticus rather than by a single seizure, and such individuals are more likely to be referred to the emergency room. For example, Nass et al. reported that most patients in their research experienced only single seizures. However, CK level was highest among patients experiencing 2 seizures in one hour. Additionally, the type of tonic–clonic seizures could play a role. Violent contraction of both the upper and lower limbs can be more frequently associated with post-ictal CK elevations. Accordingly, different muscle injuries after a fall, infection which may have triggered the seizures, and recent strenuous sportive activity could all potentially render an increase in CK levels which would blur the marginal difference present.

Despite our comprehensive literature search and careful data extraction, limitations exist in this review. As such, standardized timing for blood sampling may be difficult to achieve as the patient’s management is different in the various types of epilepsy. In addition, a limitation found in all studies is that they rely on a diagnosis of seizure in the absence of a gold-standard approach. So, the diagnosis is mainly based on history taking, which prompts potential biomarkers to be interpreted in conjunction with its clinical correlation. Further meta-analysis is considered once additional homogenous studies become available as they would explore the variation in different subtypes of CK or any other potential diagnostic marker.

## Conclusion

CK is a well-known biomarker to distinguish epileptic seizures from other causes of transient loss of consciousness, especially Syncope and PNES. The conducted retrospective study and the meta-analysis showed a significant elevation in CK after 48 h in epileptic seizures. On the other hand, CK elevation could occur in response to any form of non-specific muscle injury. The lack of specificity of CK elevation needs a thorough interpretation in the clinical setting. The low cost and the almost worldwide availability make the CK a valuable diagnostic tool to help distinguish seizures from other forms of transient loss of consciousness. Detection of high levels of CK could be potentially useful in accordance with a detailed report of the event that could be used to capture the most likely episode present in the patient. As episodes of syncope are usually not associated with muscle contraction, the presence of increased levels of CK would be favorable to other differential diagnoses.

### Supplementary Information

**Additional file 1: Table S1**. Search strategy. **Table S2**. Characteristics of included studies in meta-analysis, in epilepsy and PNES groups. **Table S3**. Characteristics of included studies in meta-analysis, in syncope and control groups. **Table S4**. Characteristics of included studies in systematic review. **Table S5**. Quality assessment of the included studies according to the National Institute of Health (NIH) quality assessment tool for observational cohort and cross-sectional studies. **Table S6**. the criteria of National Institute of Health (NIH) quality assessment tool for observational cohort and cross-sectional studies. **Table S7**. Quality assessment of the included studies according to Newcastle-Ottawa quality Scale (NOS) quality assessment tool for case control studies. **Table S8**. Subgroup analysis by age for ES and PNES groups. **Table S9**. Subgroup analysis by country for GTCS vs control group. **Table S10**. Subgroup analysis by country for different types of epileptic seizures (ES) vs control group. **Table S11**. Subgroup analysis by age and country for GTCS and syncope groups. **Table S12**. Mean CK level in GTCS patients in the first post-ictal day. **Table S13**. Mean CK level in GTCS patients in the 2nd post-ictal day. **Table S14.** Subgroup analysis by age, country, and device for single arm GTCS at 0-6 hrs following the seizure. Table 15 Subgroup analysis by age and country for single arm 1 day post ictal. **Table S16**. Subgroup analysis by age and country for single arm 2 days post ictal.

**Additional file 2: Fig. S1**. CK level in GTCS vs PNES patients (a), and sensitivity analysis of GTSC vs PNES (after removing Petramfar et al. 2009) (b). **Fig. S2**. CK level in different types of epileptic seizures vs PNES patients (a), and Subgroup analysis by mean age for different types of epileptic seizures vs PNES (b). **Fig. S3**. CK level in GTCS vs healthy controls (a), sensitivity analysis of GTCS vs healthy control (after removing Belton et al. 1967) (b), and subgroup analysis by country for GTCS vs Control (c). **Fig. S4**. CK level in different types of seizures vs healthy controls (a), sensitivity analysis different types of epileptic seizures vs healthy control (after removing Belton et al. 1967) (b), and after removing Ijaz et al. 2020 (c), and subgroup analysis by country for different types of epileptic seizures vs healthy controls (d). **Fig. S5**. CK level in GTCS vs syncope patients (a), subgroup analysis by country (b) and Subgroup analysis by age for GTCS vs Syncope (c). **Fig. S6**. Single arm analysis of mean CK level in GTCS at 0-6 hrs & day 1-2 post-ictally (a), and mean CK level in GTCS subgrouped at day 3,4,5,6,7, and unknown time of measurement (b). **Fig. S7**. Subgroup analysis by mean age (a), country (b), and device of CK measurement (c) for mean CK level in GTCS patients at the first 0-6 hrs after seizure. **Fig. S8**. Subgroup analysis by age (a), and country (b) for mean CK level in GTCS patients at day 1 post-ictally. **Fig. S9**. Subgroup analysis by mean age (a) and country (b) for mean CK level in GTCS patients at day 2 post-ictally. **Fig. S10**. leave-one-out analysis for mean CK level in GTCS patients during the first 6 hours following the seizure.

## Data Availability

The data that support the findings of this study are available from the corresponding author, [R.A.], upon reasonable request.

## Abbreviations

CK: Creatine kinase
GTCS: Generalized tonic–clonic seizures
PNES: Psychogenic non-epileptic seizures
EEG: Electroencephalography
VEM: Video electroencephalography
AUC: Area under the curve
MeSH: Medical subject headings terms
PRISMA: Preferred Reporting Items for Systematic Reviews and Meta-Analyses
NOS: Newcastle–Ottawa Quality Assessment Scale
NIH: National Institute of Health
PPV: Positive predictive value
NPV: Negative predictive value
