# Supplementary material for: The role of creatine kinase in distinguishing generalized tonic–clonic seizures from psychogenic non-epileptic seizures (PNES) and syncope: a retrospective study and meta-analysis of 1300 patients
Source: Neurol Res Pract. 2023 Oct 12;5:56. doi: 10.1186/s42466-023-00286-0 (PMC10568853; doi:10.1186/s42466-023-00286-0)
Supplement: Supplementary file 1 — Additional file 1: Table S1. Search strategy. Table S2. Characteristics of included studies in meta-analysis, in epilepsy and PNES groups. Table S3. Characteristics of included studies in meta-analysis, in syncope and control groups. Table S4. Characteristics of included studies in systematic review. Table S5. Quality assessment of the included studies according to the National Institute of Health (NIH) quality assessment tool for observational cohort and cross-sectional studies. Table S6. the criteria of National Institute of Health (NIH) quality assessment tool for observational cohort and cross-sectional studies. Table S7. Quality assessment of the included studies according to Newcastle-Ottawa quality Scale (NOS) quality assessment tool for case control studies. Table S8. Subgroup analysis by age for ES and PNES groups. Table S9. Subgroup analysis by country for GTCS vs control group. Table S10. Subgroup analysis by country for different types of epileptic seizures (ES) vs control group. Table S11. Subgroup analysis by age and country for GTCS and syncope groups. Table S12. Mean CK level in GTCS patients in the first post-ictal day. Table S13. Mean CK level in GTCS patients in the 2nd post-ictal day. Table S14. Subgroup analysis by age, country, and device for single arm GTCS at 0-6 hrs following the seizure. Table 15 Subgroup analysis by age and country for single arm 1 day post ictal. Table S16. Subgroup analysis by age and country for single arm 2 days post ictal. [file 42466_2023_286_MOESM1_ESM.docx]

**Tables Index**

**Supplemental Table 1** - Search strategy.

**Supplemental Table 2** - Characteristics of included studies in meta-analysis, in epilepsy and PNES groups.

**Supplemental Table 3** - Characteristics of included studies in meta-analysis, in syncope and control groups.

**Supplemental Table 4** - Characteristics of included studies in systematic review.

**Supplemental Table 5** - Quality assessment of the included studies according to the National Institute of Health (NIH) quality assessment tool for observational cohort and cross-sectional studies.

**Supplemental Table 6** - the criteria of National Institute of Health (NIH) quality assessment tool for observational cohort and cross-sectional studies.

**Supplemental Table 7** - Quality assessment of the included studies according to Newcastle-Ottawa quality Scale (NOS) quality assessment tool for case control studies.

**Meta-analysis Results tables (8-16)**:

**Supplemental Table 8** - Subgroup analysis by age for ES and PNES groups.

**Supplemental Table 9** - Subgroup analysis by country for GTCS vs control group.

**Supplemental Table 10** - Subgroup analysis by country for different types of epileptic seizures (ES) vs control group.

**Supplemental Table 11** - Subgroup analysis by age and country for GTCS and syncope groups.

**Supplemental Table 12 -** Mean CK level in GTCS patients in the first post-ictal day.

**Supplemental Table 13 -** Mean CK level in GTCS patients in the 2^nd^ post-ictal day.

**Supplemental Table 14 -** Subgroup analysis by age, country, and device for single arm GTCS at 0-6 hrs following the seizure.

**Supplemental Table 15 -** Subgroup analysis by age and country for single arm 1 day post ictal.

**Supplemental Table 16 -** Subgroup analysis by age and country for single arm 2 days post ictal.

**Supplemental Table 1. Search strategy**

| **Database** | **Search was carried out by** | **Term** |
| --- | --- | --- |
| **PubMed** | title and abstract | 1. (Seizure OR seizures OR pseudo-seizures OR pseudo-attacks OR convulsions OR epilepsy OR epilepsies OR aura OR "non-epileptic events" OR " psychogenic attacks" OR "non-epileptic attacks") 2. ("Creatine Kinase" OR "ATP Creatine Phosphotransferase" OR "Creatine Phosphokinase" OR "ADP Phosphocreatine Phosphotransferase" OR "CPK" OR "CK" OR "creatine kinase" OR "Macro-Creatine Kinase" OR "Macro Creatine Kinase"). 3. 1 AND 2 |
| **Scopus** |  |  |
| **Web Of Science** | topic |  |
| **Embase** | title |  |

**Supplemental Table 2. Characteristics of included studies in meta-analysis, in epilepsy and PNES groups.**

| Author | **Year** | **Country** | **Study Design** | **Time of CK measurement** | **Basal CK value (mU/mL)** | **Epileptic seizure** | | | | **PNES** | | |
| --- | --- | --- | --- | --- | --- | --- | --- | --- | --- | --- | --- | --- |
|  |  |  |  |  |  | **No of patients** | **Age, mean (SD** | **sex (M:F)** | **Type of ES** | **No of patients** | **Age, mean (SD)** | **sex (M:F)** |
| Current study. | **2022** | Germany | Retrospective cohort | From day one of admission till day seven | NR | 151 | 56.5(21.2) | 83:68 | NR | - | - | - |
| Ijaz et al. [9] | **2020** | USA | Case control | 30 min after the attack | NR | 30 | 21.6 (4.1) | 0:30 | grand mal, GTCS | 30 | 21.9 (5.5) | 0:30 |
| Barbella et al. [10] | **2020** | Switzerland | Retrospective cohort | median 1.7h to 2h | 190 mU\mL | 75 | 47.5(19.6) | 46:29 | FTCS | **-** | **-** | **-** |
| Nass et al. [11] | **2019** | Germany | Prospective cohort | at baseline, prior to occurrence of TCS, within 30min, after 2 ,6, 24 and 48 hrs after TCS | NR | 32 | 33.46(12.13) | 15:17 | GTCS and FBTCS | **-** | **-** | **-** |
| Dafotakis et al. [12] | **2018** | Germany | Case control | 2 phases (samples): first was within 2 hrs after the event and the second was 10-48h after event | NR | 30 | 49.5(20.5) | 17:13 | GTCS | **-** | **-** | **-** |
| Javali et al. [13] | **2017** | India | Case control | 0-5 hrs after attack | NR | 91 | over 15 years |  | GTCS focal, status | 9 |  |  |
| Matz et al. [14] | **2017** | Germany | Case control | ranging from 0-120 min | 170 mU/mL | 49 | 47.0 (21.0) | 32:17 | NR | **-** | **-** | **-** |
| Petramfar et al. [15] | **2009** | Iran | Case control | 12-15 hrs after attack for the epilepsy group | NR | 20 | 39.5 (21.063) | 6:14 | GTCS | 20 | 38.95(14.573) | 11:9 |

| Willert et al. [16] | **2004** | Germany | Case control | 7 times from 10min till 24h (besides baseline) | women, the normal limit is <168mU/mL, for men <195mU/mL | 32 | 33.6 (10.8) | 21:11 | 2ry GTCS, focal | 12 | 37.8 (13.1) | 6:6 |
| --- | --- | --- | --- | --- | --- | --- | --- | --- | --- | --- | --- | --- |
| Finsterer et al. [17] | **2000** | Vienna | Retrospective cohort | NR | 70mU/mL | 58 | 54.7 | 37:21 | NR | **-** | **-** | **-** |
| Neufeld et al. [18] | **1997** | Israel | Case control | few hours from attack (at admission) and after 24 hrs (Day 2) | 130mU\mL | 16 | 31 (11) | 8:8 | GTCS | **-** | **-** | **-** |
| Libman et al. [19] | **1991** | Canada | Case control | all <1hr, 1-3hrs, >3hrs after seizure | <188mU/mL | 42 | 50.6(3) | 23:19 | TCS | **-** | **-** | **-** |
| Cheason et al. [20] | **1983** | USA | Case control | at admission, 12 and 24 h after last seizure, daily for six days | NR | 25 | 55.7(16.4) | 14:11 | GS, FMS, ARS | **-** | **-** | **-** |
| Glotzner et al. [21] | **1974** | Germany | Cross sectional | Every day from admission till 7th post ictal day | upper normal limit 50mU/mL | 17 | 48(36.7) | 14:3 | GS | **-** | **-** | **-** |
| Goto et al. [22] | **1974** | Japan | Case control | **-** | NR | 5 | **-** | **-** | GS | **-** | **-** | **-** |
| Belton et al. [23] | **1967** | USA | Case control | **-** | 30.8 mU/mL (±11.5), and the upper limit of normal (2 S.D.’s) was 54.1 | 60 | Children | **-** | grand mal, psychomotor, focal | **-** | **-** | **-** |

GS: generalized seizure, GTCS: generalized tonic clonic seizure, FBTCS: focal bilateral tonic clonic seizure, FMS: focal motor seizure, ARS: alcohol related seizure

**Supplemental Table 3. Characteristics of included studies in meta-analysis, in syncope and control groups.**

| study | Year | Syncope | | | | Healthy Controls | | |  |
| --- | --- | --- | --- | --- | --- | --- | --- | --- | --- |
|  |  | **No of patients** | **Age, mean (SD)** | **sex (M:F)** | **Type of syncope** | **No of patients** | **Age, mean (SD)** | **sex (M:F)** |  |
| Current study. | 2022 | 51 | 67.3(20) | 26:25 | NR | - | - | - |  |
| Ijaz et al. [9] | 2020 | **-** | **-** | **-** | **-** | 30 | 23.4(4.7) | 0:30 |  |
| Barbella et al. [10] | 2020 | **-** | **-** | **-** | **-** | **-** | **-** | **-** |  |
| Dafotakis et al. [12] | 2018 | 15 | 65.1(16.3) | 10:5 | vasovagal, orthostatic, infection associated, cardiogenic syncope, non-ST elevation myocardial infarction and etiologically unclear syncopes. | **-** | **-** | **-** |  |
| Matz et al. [14] | 2017 | 36 | 59.5 (21.4) | 24:12 | NR | **-** | **-** | **-** |  |
| Petramfar et al. [15] | 2009 | 22 | 39.36(14.178) | 11:11 | vasovagal attack. | 20 | 43.20 (15.793) | 12:8 |  |
| Willert et al. [16] | 2004 | **-** | **-** | **-** | **-** | 16 | 34.9(9.2) | 8:8 |  |
| Neufeld et al. [18] | 1997 | 17 | 32(13) | 9:8 | vasovagal attack. | **-** | **-** | **-** |  |
| Libman et al. [19] | 1991 | 54 | 57(2.7) | 25:29 | symptomatic cardiac arrhythmia, vasodepressor episode, head trauma and Unclear diagnosis. | **-** | **-** | **-** |  |
| Belton et al. [23] | 1967 | **-** | **-** | **-** | **-** | 21 | children | **-** |  |

**Supplemental Table 4. Characteristics of included studies in systematic review.**

| Author | Year | Country | Study Design | Aim | Conclusion |
| --- | --- | --- | --- | --- | --- |
| Goksu  et al. [24] | **2009** | Turkey | Prospective cohort | to differentiate patients who have tonic–clonic seizures from patients with syncopal attacks using serum creatine kinase and myoglobin levels in an acceptable observation period for patients presenting to the ED. | Serum creatine kinase measured at the fourth hour of loss of consciousness may be a potentially useful laboratory test to differentiate tonic–clonic seizure from syncope. Patently, it requires and warrants further study. |
| Finsterer et al. [25] | **2004** | Vienna | Retrospective cohort | In how many patients with idiopathic CK-elevation of this previous study did CK remain elevated, in how many patients the cause of previous idiopathic CK-elevation could be determined in retrospective; and which are the most frequent causes of previously idiopathic CK-elevation. | Though CK-elevation persists until follow-up in only one third of these patients, the cause of previous CK elevation can be determined in half of them at follow-up. The most frequent causes of previously idiopathic CK-elevation at follow-up are neuromuscular disorder, stroke, and seizure. |
| Wyllie  et al. [26] | **1985** | USA | Prospective cohort | to use the highly controlled setting of the EEG laboratory to determine the specificity and sensitivity of postictal CK elevation as a marker for recent epileptic seizures. | postictal CK determination can serve as an adjunctive test for differentiation between psychogenic and epileptic generalized tonoclonic seizures. |

**Supplemental Table 5. Quality assessment of the included studies according to the National Institute of Health (NIH) quality assessment tool for observational cohort and cross-sectional studies.**

| Study | Year | C1 | C2 | C3 | C4 | C5 | C6 | C7 | C8 | C9 | C10 | C11 | C12 | C13 | C14 | Total score |
| --- | --- | --- | --- | --- | --- | --- | --- | --- | --- | --- | --- | --- | --- | --- | --- | --- |
| Current study. | **2022** | YES | YES | YES | YES | YES | YES | NO | NO | YES | NO | YES | NO | YES | NO | **9** |
| Barbella et al. [10] | **2020** | YES | NO | YES | YES | NO | NO | YES | NO | YES | NO | YES | NO | YES | NO | **7** |
| Nass et al. [11] | **2019** | YES | NO | NO | YES | NO | YES | YES | NO | YES | NO | YES | YES | NO | YES | **8** |
| Dafotakis et al. [12] | **2018** | YES | YES | YES | YES | NO | YES | YES | NO | YES | NO | YES | NO | YES | NO | **9** |
| Javali et al. [13] | **2017** | YES | YES | YES | YES | NO | NO | YES | NO | YES | YES | YES | NO | YES | NO | **9** |
| Matz et al. [14] | **2017** | YES | YES | YES | YES | NO | NO | YES | NO | YES | YES | YES | NO | YES | NO | **9** |
| Goksu et al. [24] | **2009** | YES | YES | YES | YES | NO | YES | NO | YES | YES | YES | NO | NO | NO | NO | **8** |
| Finsterer et al. [25] | **2004** | YES | YES | NO | YES | NO | NO | YES | NO | NO | NO | YES | NO | NO | NO | **5** |
| Finsterer et al. [17] | **2000** | YES | YES | NO | YES | NO | NO | YES | NO | NO | NO | YES | NO | YES | NO | **6** |
| Wyllie et al. [26] | **1985** | YES | YES | YES | YES | NO | YES | YES | NO | YES | NO | YES | NO | NO | NO | **7** |
| Glötzner et al. [21] | **1974** | YES | NO | YES | YES | NO | YES | YES | NO | YES | NO | YES | NO | YES | NO | **8** |

**Supplemental Table 6. the criteria of National Institute of Health (NIH) quality assessment tool for observational cohort and cross-sectional studies.**

| Was the research question or objective in this paper clearly stated? | Criterion 1 |
| --- | --- |
| Was the study population clearly specified and defined? | Criterion 2 |
| Was the participation rate of eligible persons at least 50%? | Criterion 3 |
| Criterion 4 Were all the subjects selected or recruited from the same or similar populations (including the same time period)? Were inclusion and exclusion criteria for being in the study prespecified and applied uniformly to all participants? | Criterion 4 |
| Was a sample size justification, power description, or variance and effect estimates provided? | Criterion 5 |
| For the analyses in this paper, were the exposure(s) of interest measured prior to the outcome(s) being measured? | Criterion 6 |
| Was the timeframe sufficient so that one could reasonably expect to see an association between exposure and outcome if it existed? | Criterion 7 |
| For exposures that can vary in amount or level, did the study examine different levels of the exposure as related to the outcome (e.g., categories of exposure, or exposure measured as continuous variable)? | Criterion 8 |
| Were the exposure measures (independent variables) clearly defined, valid, reliable, and implemented consistently across all study participants? | Criterion 9 |
| Was the exposure(s) assessed more than once over time? | Criterion 10 |
| Were the outcome measures (dependent variables) clearly defined, valid, reliable, and implemented consistently across all study participants? | Criterion 11 |
| Were the outcome assessors blinded to the exposure status of participants? | Criterion 12 |
| Was loss to follow-up after baseline 20% or less? | Criterion 13 |
| Were key potential confounding variables measured and adjusted statistically for their impact on the relationship between exposure(s) and outcome(s)? | Criterion 14 |

**Supplemental Table 7. Quality assessment of the included studies according to Newcastle-Ottawa quality Scale (NOS) quality assessment tool for case control studies.**

| Study | Year | Selection | | | | Comparability | | Exposure | | | Score |
| --- | --- | --- | --- | --- | --- | --- | --- | --- | --- | --- | --- |
|  |  | adequate case definition? | Representativeness of the cases | Selection of Controls | Definition of Controls | Study controls for age | Study controls for  any additional factor (sex, Edu, SE, dis..) | Ascertainment of exposure | Same method of ascertainment for cases and controls | Non-Response rate |  |
| Ijaz et al. [9] | 2020 | **★** | **-** | **-** | **★** | **★** | **-** | **-** | **-** | **★** | 4 |
| Petramfar et al. [15] | 2009 | **★** | **-** | **-** | **★** | **★** | **-** | **★** | **★** | **★** | 6 |
| Willert et al. [16] | 2004 | **★** | **-** | **-** | **★** | **★** | **★** | **★** | **★** | **★** | 7 |
| Neufeld et al. [18] | 1997 | **★** | **-** | **-** | **★** | **★** | **★** | **★** | **★** | **★** | 7 |
| Libman et al. [19] | 1991 | **★** | **★** | **★** | **★** | **★** | **★** | **★** | **★** | **-** | 8 |
| Cheason et al. [20] | 1983 | **★** | **-** | **-** | **-** | **-** | **-** | **-** | **★** | **★** | 3 |
| Goto et al. [22] | 1974 | **-** | **-** | **-** | **-** | **-** | **-** | **-** | **★** | **★** | 2 |
| Belton et al. [23] | 1967 | **★** | **-** | **-** | **★** | **★** | **-** | **★** | **★** | **★** | 6 |

**Meta-analysis Results tables:**

**Supplemental Table 8. Subgroup analysis by age for epileptic seizures and PNES group**

|  | epileptic seizures vs PNES sub-groups results | | | | |
| --- | --- | --- | --- | --- | --- |
| Subgroup | **Studies** | **MD (mIU\ml)** | **(95%C.I)** | **I²** | **p-value** |
| Age > 40 years | 1 | 820.28 | (669.59, 970.967) | - | - |
| Age < 40 years | 3 | 473.16 | (85.12, 861.20) | 97.11% | = 0 |

**Supplemental Table 9. Subgroup analysis by country for GTCS vs control group**

|  | GTCS vs Control | | | | |
| --- | --- | --- | --- | --- | --- |
| Subgroup (country) | **Number of Studies** | **MD (mIU\ml)** | **(95%C.I)** | **I²** | **p-value** |
| USA | 2 | 913.09 | (-779.53, 2605.71) | 99.84% | = 0 |
| Iran | 1 | 190 | (84.36, 295.64) | NA | NA |

**Supplemental Table 10. Subgroup analysis by country for different types of epileptic seizures vs control group**

|  | GTCS VS Control | | | | |
| --- | --- | --- | --- | --- | --- |
| Subgroup (country) | **Number of Studies** | **MD (mIU\ml)** | **(95%C.I)** | **I²** | **p-value** |
| Germany | 1 | 75.94 | (20.09, 131.80) | NA | NA |
| USA | 2 | 907.59 | (-795.81, 2611.00) | 99.85% | = 0 |
| Iran | 1 | 190 | (84.36, 295.64) | NA | NA |

**Supplemental Table 11. Subgroup analysis by age and country for GTCS and syncope groups**

|  | GTCS vs syncope | | | | |
| --- | --- | --- | --- | --- | --- |
| Subgroup | **Number of Studies** | **MD (mIU\ml)** | **(95% C.I)** | **I²** | **p-value** |
| Day of measurements |  |  |  |  |  |
| Day 1 | **6** | **117.77** | **(28.71, 206.83)** | **88.79%** | **= 0** |
| Day 2 | 2 | 175.96 | (39.97, 311.95) | 0% | = 0.74 |
| Age |  |  |  |  |  |
| Age > 40 years | 4 | 178.67 | (19.96, 337.39) | 91.09%, | = 0 |
| Age < 40 years | 2 | 156.16 | (76.87, 235.44) | 1.08% | = 0.03 |
| Country |  |  |  |  |  |
| Germany | 3 | 199.12 | (-48.25, 446.48) | 93.28% | = 0 |
| Canada | 1 | 160.60 | (91.52, 119.68 | NA | NA |
| Iran | 1 | 192 | (86.72, 297.28) | NA | NA |
| Israel | 1 | 110.5 | (-8.48, 229.48) | NA | NA |

**Supplemental Table 12. Mean CK level in GTCS patients in the first post-ictal day**

|  | Mean CK level in GTCS patients (day 1-2) | | | | |
| --- | --- | --- | --- | --- | --- |
| Subgroup | **Number of Studies** | **MD (mIU\ml)** | **(95%C.I)** | **I²** | **Heterogeneity p-value** |
| 0-6 hours post-ictal | 9 | 721.6 | (403.75, 1039.44) | 99.75% | = 0 |
| 24 hours post-ictal | 5 | 207.55 | (117.38, 297.71) | 87.82% | = 0 |
| Day 2 post-ictal | 5 | 249.71 | (143.4, 356.02) | 34.8% | = 0.19 |

**Supplemental Table 13. Mean CK level in GTCS patients in the second post-ictal day**

|  | Mean CK level in GTCS patients (≥ 3 days) | | | | |
| --- | --- | --- | --- | --- | --- |
| Subgroup | **Number of Studies** | **MD (mIU\ml)** | **(95%C.I)** | **I²** | **p-value** |
| Day 3 post-ictal | 3 | 577.8 | (-221.07, 1376.67) | 53.33% | = 0.12 |
| Day 4 post-ictal | 3 | 359.65 | (-34.18, 753.49) | 17.82% | = 0.3 |
| Day 5 post-ictal | 3 | 738.55 | (252.43, 1224.67) | 59.15% | = 0.09 |
| Day 6 post-ictal | 3 | 744.49 | (63.22, 1425.77) | 77.98% | = 0.01 |
| Day 7 post-ictal | 2 | 156.48 | (115.65, 197.31) | 0% | = 0.43 |
| Unknown time | 2 | 215.15 | (92.71, 337.59) | 74.7% | = 0.05 |

**Supplemental Table 14. Subgroup analysis by age, country, and device for single arm GTCS at 0-6 hrs following the seizure**

|  | Mean CK level in GTCS patients 0-6 hours. | | | | |
| --- | --- | --- | --- | --- | --- |
| Subgroup | **Studies** | **MD (mIU\ml)** | **(95% C.I)** | **I²** | **p-value** |
| Age |  |  |  |  | |
| Age > 40 years | 6 | 570.17 | (360.98, 771.37) | 97.86% | = 0 |
| Age < 40 years | 3 | 928.66 | (72.28, 1785.04) | 99,93% | = 0 |
| Country | | | | |  |
| Germany | 3 | 170.80 | (64.99, 276.60) | 92.08% | = 0 |
| USA | 2 | 1361.39 | (-1020.45, 3743.24) | 99.96% | = 0 |
| Canada | 1 | 231.10 | (162.89, 299.31) | NA | NA |
| Israel | 1 | 116 | (79.25, 125.75) | NA | NA |
| India | 1 | 1379.3 | (1203.72, 1554.88) | NA | NA |
| Switzerland | 1 | 1488 | (1124.59, 1852.41) | NA | NA |
| Device | | | | |  |
| Boehringer, Mannheim | 2 | 236.36 | (165.76, 302.96) | 0% | = 0.488 |
| Automatic Analyzer | 1 | 146.50 | (114.04, 178.96) | NA | NA |
| Undefined | 3 | 387.84 | (194.08, 581.61) | 96.8% | = 0 |
| Dimension | 2 | 735.62 | (-519.89, 1991.14) | 99.5% | = 0 |
| Randox Kit | 1 | 2577 | (2488.97; 2665.03) | NA | NA |

**Supplemental Table 15. Subgroup analysis by age and country for single arm 1 day post ictal**

|  | Single arm 1 day post ictal | | | | |
| --- | --- | --- | --- | --- | --- |
| Subgroup | **Studies** | **MD** | **(95% C.I)** | **I²** | **p-value** |
| Age | | | | | |
| Age > 40 years | 4 | 334.8 | (93.31, 576.29) | 90.46% | = 0 |
| Age < 40 years | 1 | 109.03 | (80.57, 137.49) | NA | NA |
| Country | | | | | |
| Germany | 4 | 201.93 | (113.39, 290.47) | 90.15% | = 0 |
| USA | 1 | 1324.92 | (-200.36; 2850.20) | 69.34% | = 0.07 |

**Supplemental Table 16. Subgroup analysis by age and country for single arm 2 days post ictal**

| Single arm 2 days post ictal | | | | | |
| --- | --- | --- | --- | --- | --- |
| Subgroup | **Number of Studies** | **MD (mIU\ml)** | **(95% C.I)** | **I²** | **p-value** |
| Age | | | | | |
| Age > 40 years | 3 | 350.13 | (178.45, 521.81) | 0% | = 0.42 |
| Age < 40 years | 2 | 196.48 | 92.94, 300.02) | 34.17% | = 0.22 |
| Country | | | | | |
| Germany | 3 | 214.59 | (80.02, 349.17) | 31.49% | = 0.23 |
| USA | 1 | 472.55 | (128.63, 816.47) | NA | NA |
| Israel | 1 | 271.00 | (116.65, 425.35) | NA | NA |
