# Supplementary material for: The role of creatine kinase in distinguishing generalized tonic–clonic seizures from psychogenic non-epileptic seizures (PNES) and syncope: a retrospective study and meta-analysis of 1300 patients
Source: Neurol Res Pract. 2023 Oct 12;5:56. doi: 10.1186/s42466-023-00286-0 (PMC10568853; doi:10.1186/s42466-023-00286-0)
Supplement: Supplementary file 2 — Additional file 2: Fig. S1. CK level in GTCS vs PNES patients (a), and sensitivity analysis of GTSC vs PNES (after removing Petramfar et al. 2009) (b). Fig. S2. CK level in different types of epileptic seizures vs PNES patients (a), and Subgroup analysis by mean age for different types of epileptic seizures vs PNES (b). Fig. S3. CK level in GTCS vs healthy controls (a), sensitivity analysis of GTCS vs healthy control (after removing Belton et al. 1967) (b), and subgroup analysis by country for GTCS vs Control (c). Fig. S4. CK level in different types of seizures vs healthy controls (a), sensitivity analysis different types of epileptic seizures vs healthy control (after removing Belton et al. 1967) (b), and after removing Ijaz et al. 2020 (c), and subgroup analysis by country for different types of epileptic seizures vs healthy controls (d). Fig. S5. CK level in GTCS vs syncope patients (a), subgroup analysis by country (b) and Subgroup analysis by age for GTCS vs Syncope (c). Fig. S6. Single arm analysis of mean CK level in GTCS at 0-6 hrs & day 1-2 post-ictally (a), and mean CK level in GTCS subgrouped at day 3,4,5,6,7, and unknown time of measurement (b). Fig. S7. Subgroup analysis by mean age (a), country (b), and device of CK measurement (c) for mean CK level in GTCS patients at the first 0-6 hrs after seizure. Fig. S8. Subgroup analysis by age (a), and country (b) for mean CK level in GTCS patients at day 1 post-ictally. Fig. S9. Subgroup analysis by mean age (a) and country (b) for mean CK level in GTCS patients at day 2 post-ictally. Fig. S10. leave-one-out analysis for mean CK level in GTCS patients during the first 6 hours following the seizure. [file 42466_2023_286_MOESM2_ESM.docx]

**Figures Index:**

**Supplemental Figure 1**. **Supplemental Figure 1. CK level in GTCS vs PNES patients (a), and sensitivity analysis of GTSC vs PNES (after removing Petramfar et al. 2009) (b)**

**Supplemental Figure 2. CK level in different types of epileptic seizures vs PNES patients (a), and Subgroup analysis by mean age for different types of epileptic seizures vs PNES (b)**

**Supplemental Figure 3. CK level in GTCS vs healthy controls (a), sensitivity analysis of GTCS vs healthy control (after removing Belton et al. 1967) (b), and subgroup analysis by country for GTCS vs Control (c)**

**Supplemental Figure 4. CK level in different types of seizures vs healthy controls (a), sensitivity analysis different types of epileptic seizures vs healthy control (after removing Belton et al. 1967) (b), and after removing Ijaz et al. 2020 (c), and subgroup analysis by country for different types of epileptic seizures vs healthy controls (d)**

**Supplemental Figure 5. CK level in GTCS vs syncope patients (a), subgroup analysis by country (b) and Subgroup analysis by age for GTCS vs Syncope (c)**

**Supplemental Figure 6. Single arm analysis of mean CK level in GTCS at 0-6 hrs & day 1-2 post-ictally (a), and mean CK level in GTCS subgrouped at day 3,4,5,6,7, and unknown time of measurement (b)**

**Supplemental Figure 7. Subgroup analysis by mean age (a), country (b), and device of CK measurement (c) for mean CK level in GTCS patients at the first 0-6 hrs after seizure**

**Supplemental Figure 8. Subgroup analysis by age (a), and country (b) for mean CK level in GTCS patients at day 1 post-ictally**

**Supplemental Figure 9. Subgroup analysis by mean age (a) and country (b) for mean CK level in GTCS patients at day 2 post-ictally**

**Supplemental Figure 10. leave-one-out analysis for mean CK level in GTCS patients during the first 6 hours following the seizure**

| 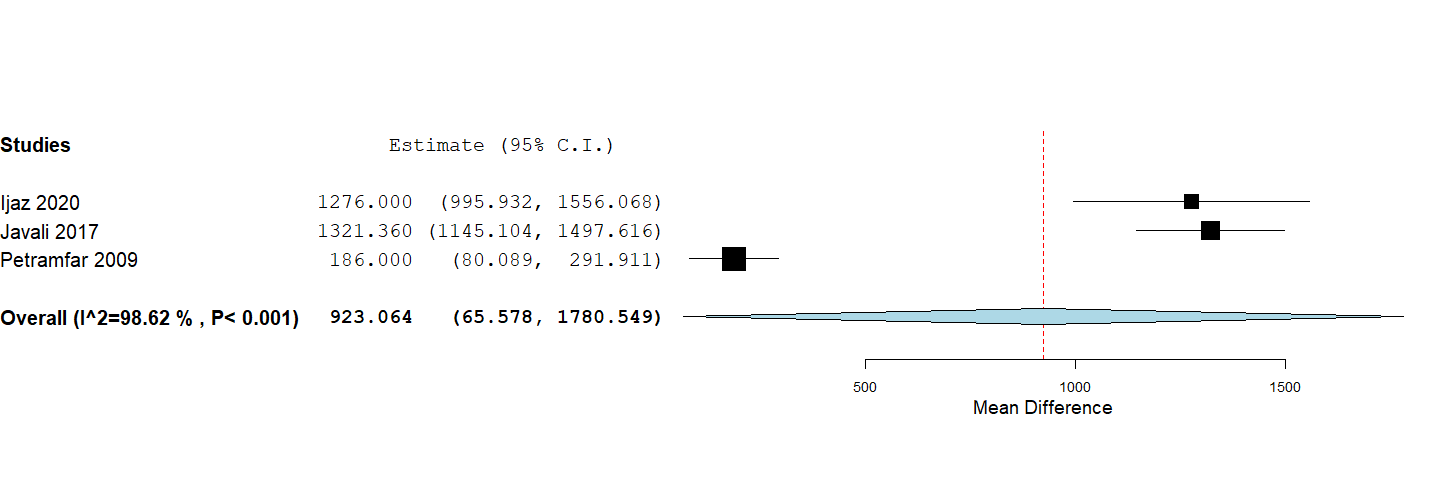 | **(a)** |
| --- | --- |
| **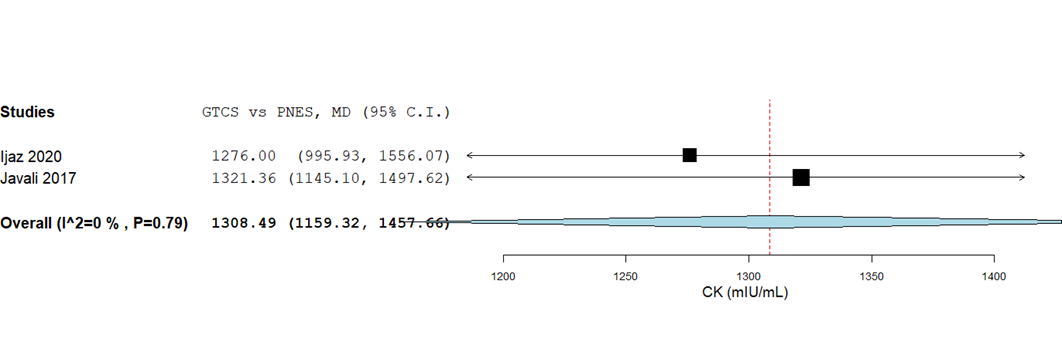** | **(b)** |

**Supplemental Figure 1. CK level in GTCS vs PNES patients (a), and sensitivity analysis of GTSC vs PNES (after removing Petramfar et al. 2009) (b)**

| 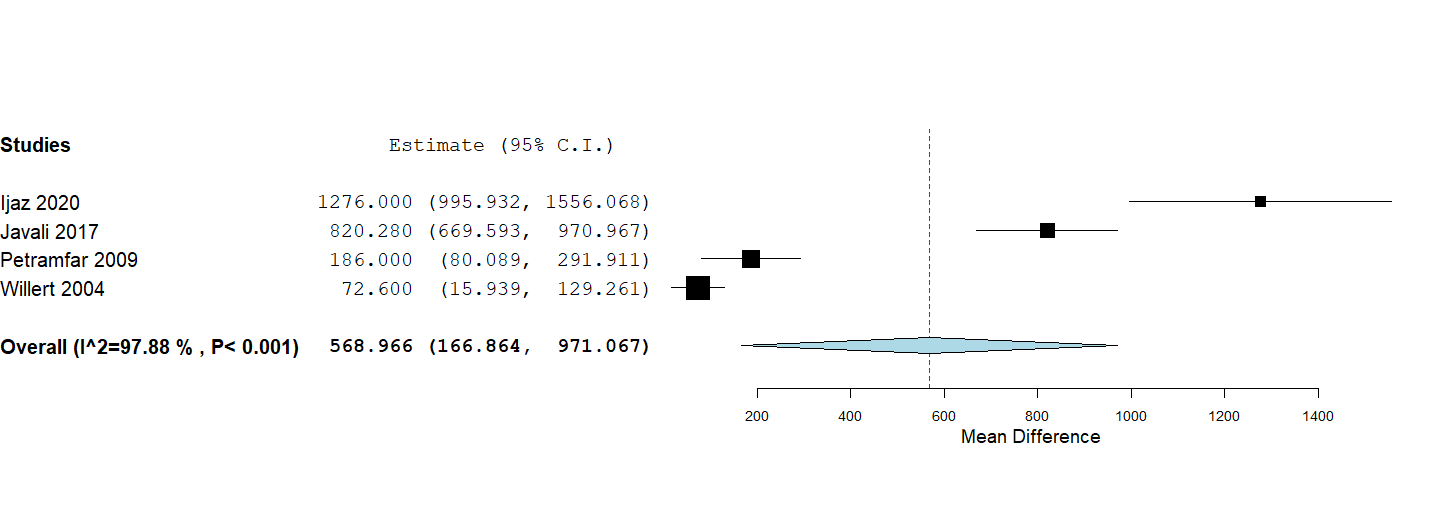 | **(a)** |
| --- | --- |
| 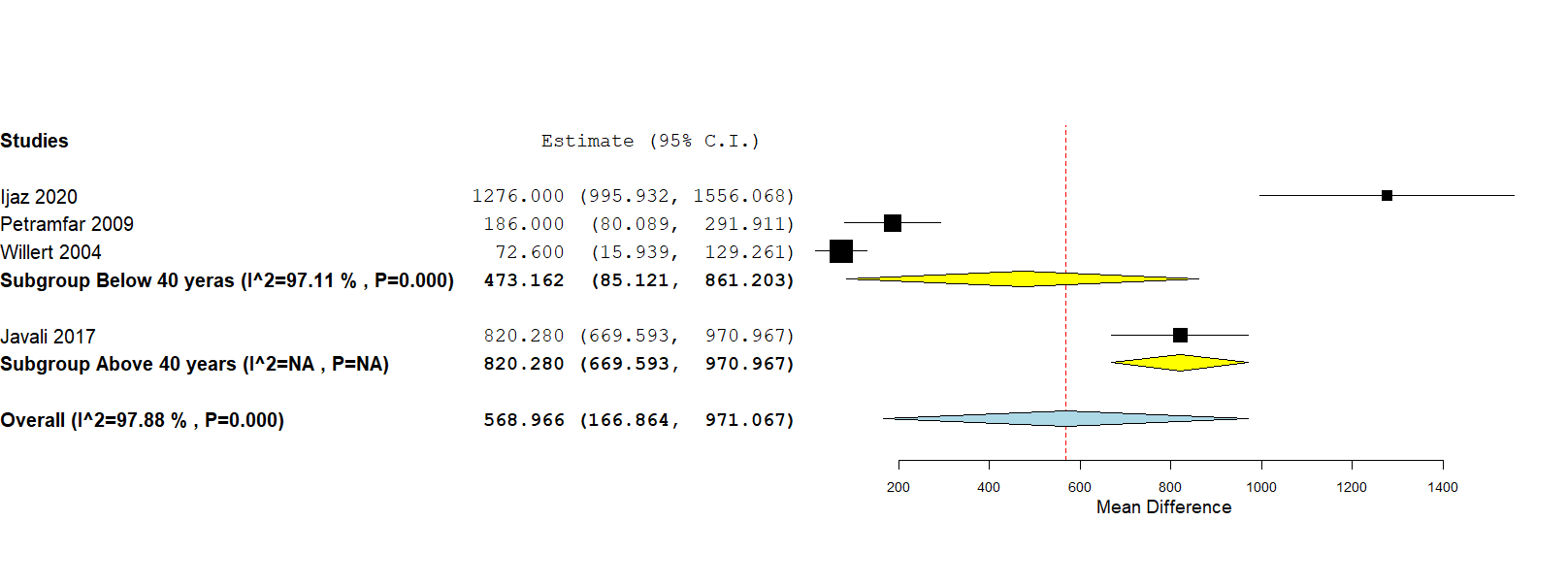 | **(b)** |

**Supplemental Figure 2. CK level in different types of epileptic seizures vs PNES patients (a), and Subgroup analysis by mean age for different types of epileptic seizures vs PNES (b)**

| **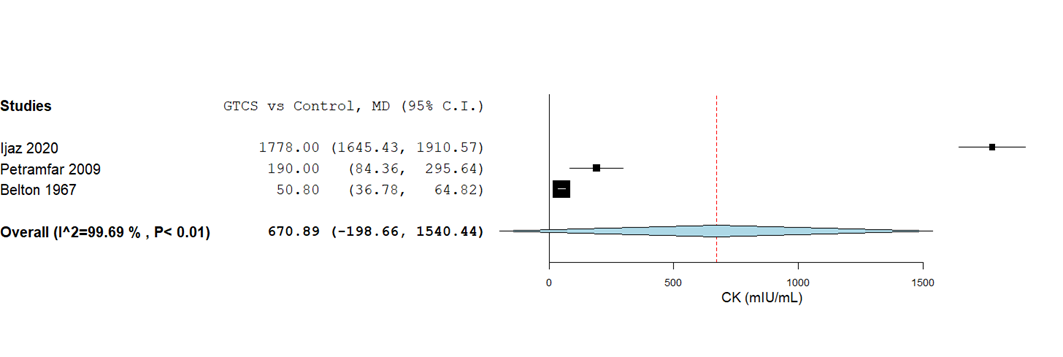** | **(a)** |
| --- | --- |
| **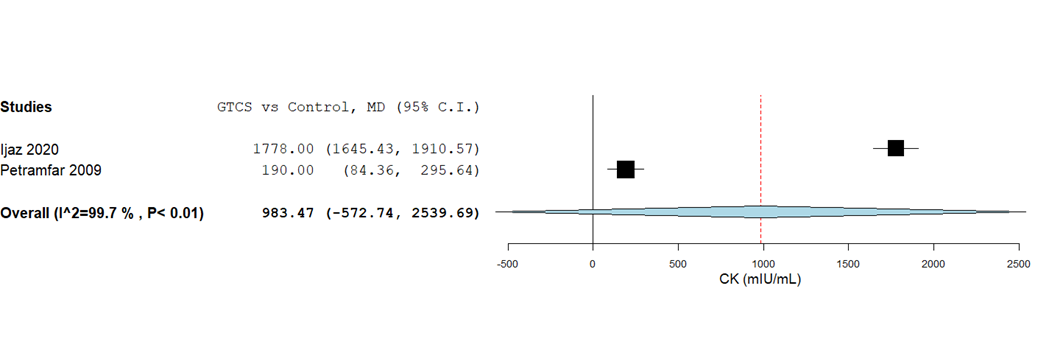** | **(b)** |
| **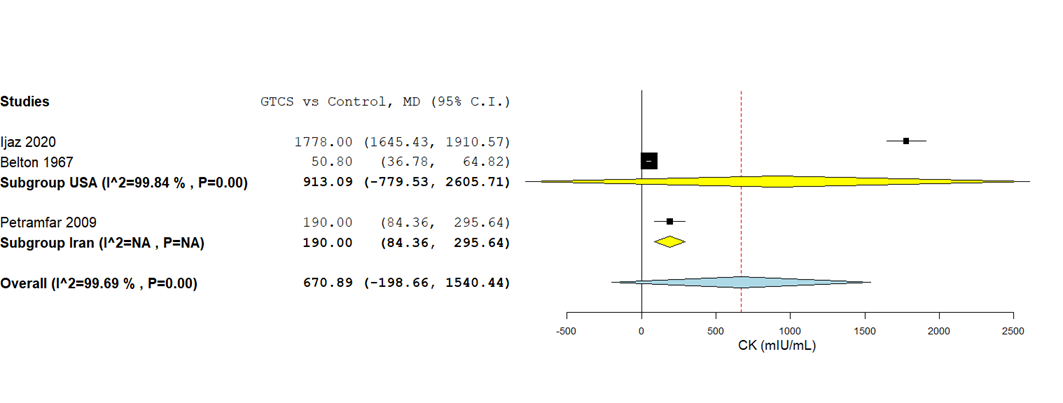** | **(c)** |

**Supplemental Figure 3. CK level in GTCS vs healthy controls (a), sensitivity analysis of GTCS vs healthy control (after removing Belton et al. 1967) (b), and subgroup analysis by country for GTCS vs Control (c)**

| **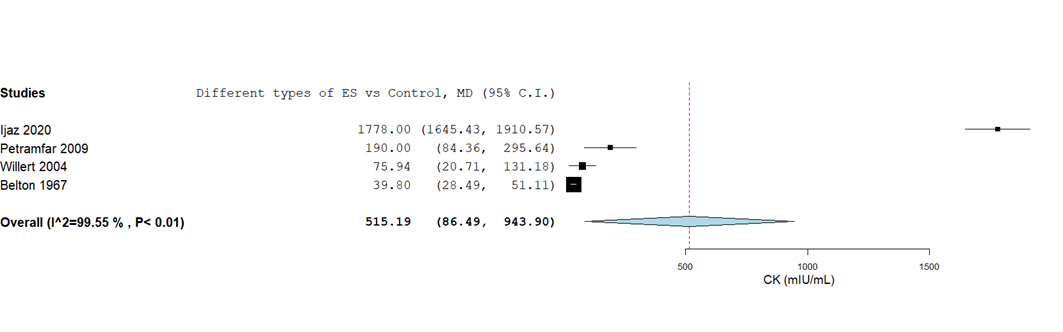** | **(a)** |
| --- | --- |
| **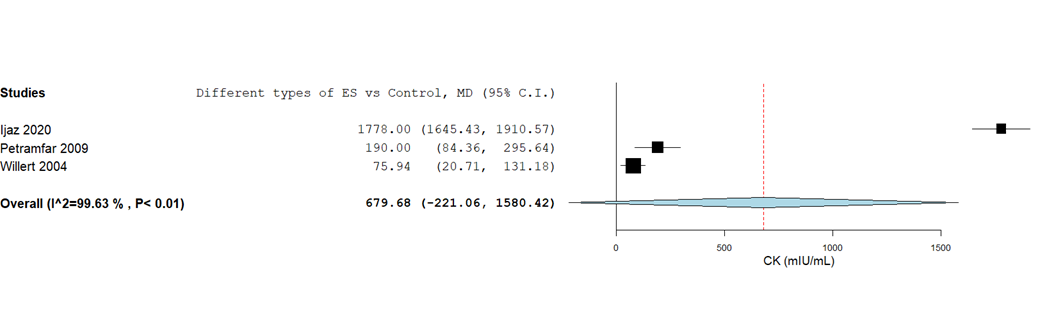** | **(b)** |
| **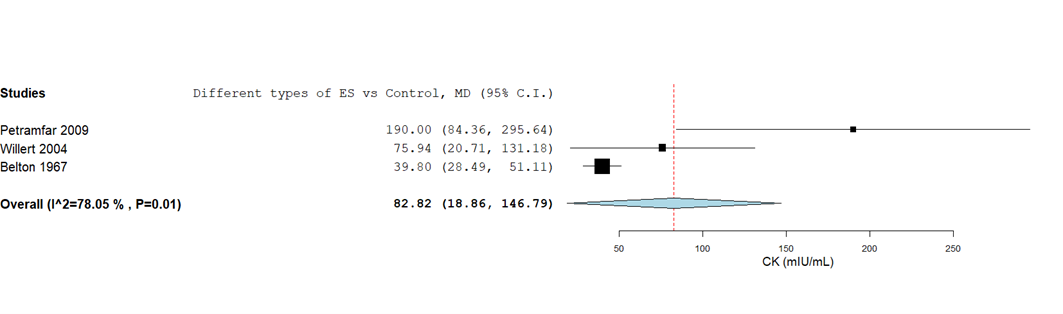** | **(c)** |
| **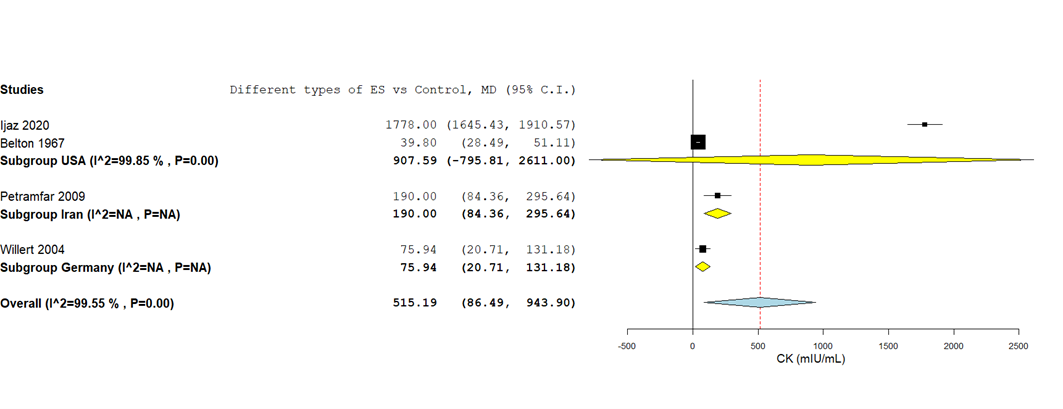** | **(d)** |

**Supplemental Figure 4. CK level in different types of seizures vs healthy controls (a), sensitivity analysis different types of epileptic seizures vs healthy control (after removing Belton et al. 1967) (b), and after removing Ijaz et al. 2020 (c), and subgroup analysis by country for different types of epileptic seizures vs healthy controls (d)**

| **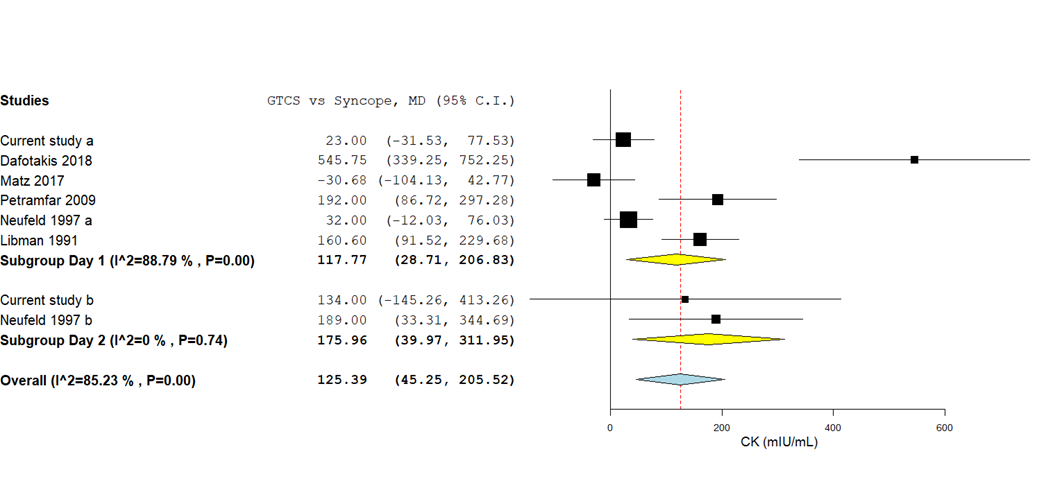** | **(a)** |
| --- | --- |
| **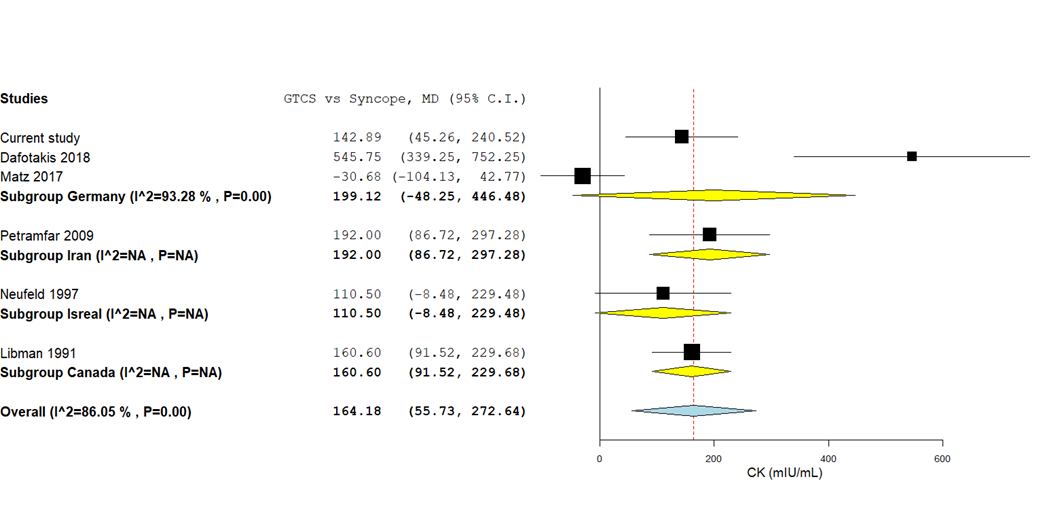** | **(b)** |
| **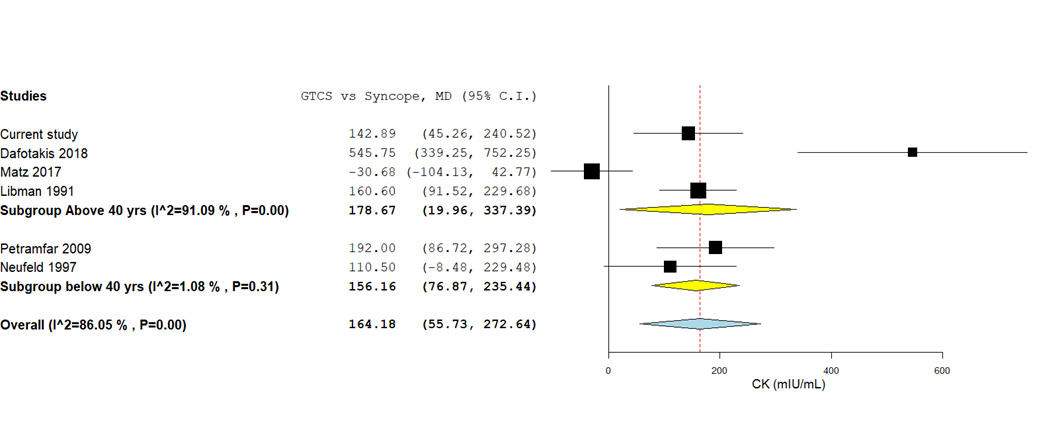** | **(c)** |

**Supplemental Figure 5. CK level in GTCS vs syncope patients (a), subgroup analysis by country (b) and Subgroup analysis by age for GTCS vs Syncope (c)**

| **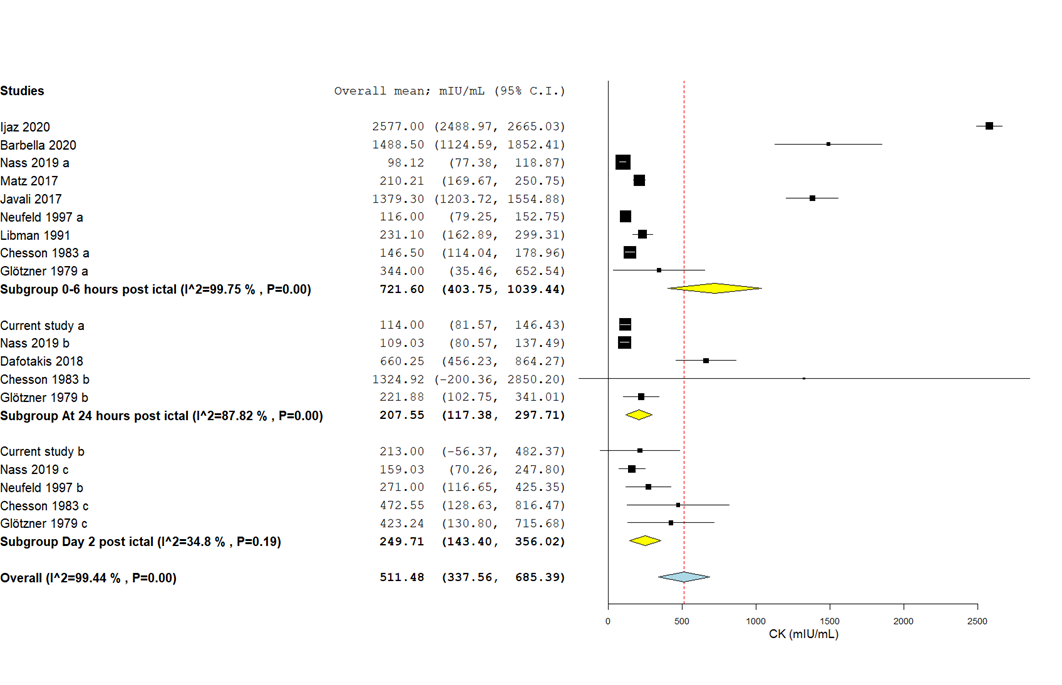** | **(a)** |
| --- | --- |
| **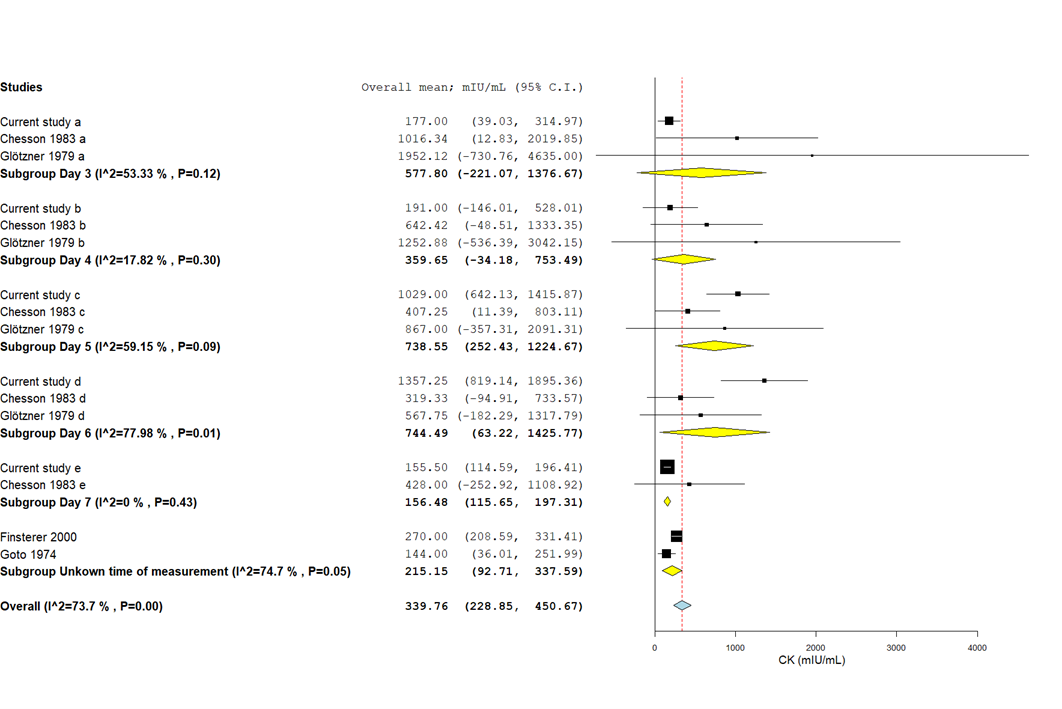** | **(b)** |

**Supplemental Figure 6. Single arm analysis of mean CK level in GTCS at 0-6 hrs & day 1-2 post-ictally (a), and mean CK level in GTCS subgrouped at day 3,4,5,6,7, and unknown time of measurement (b)**

| **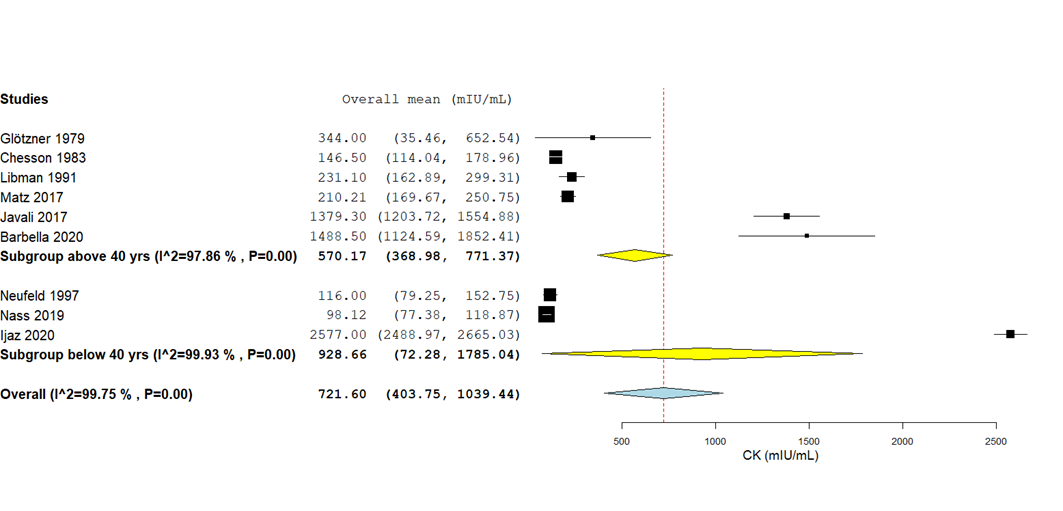** | **(a)** |
| --- | --- |
| **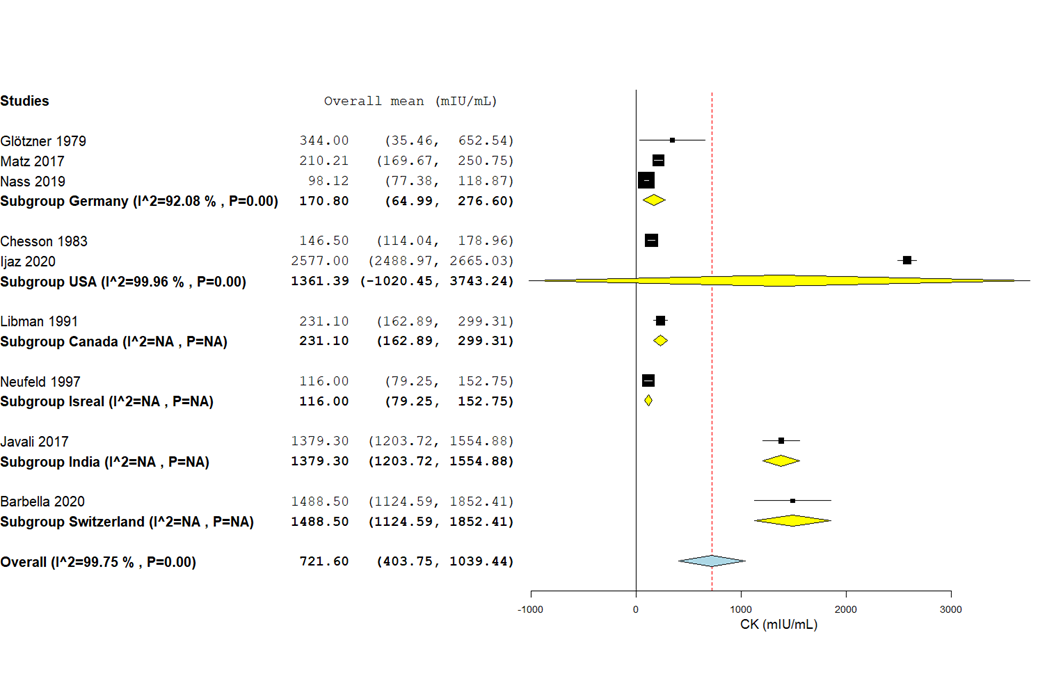** | **(b)** |
| **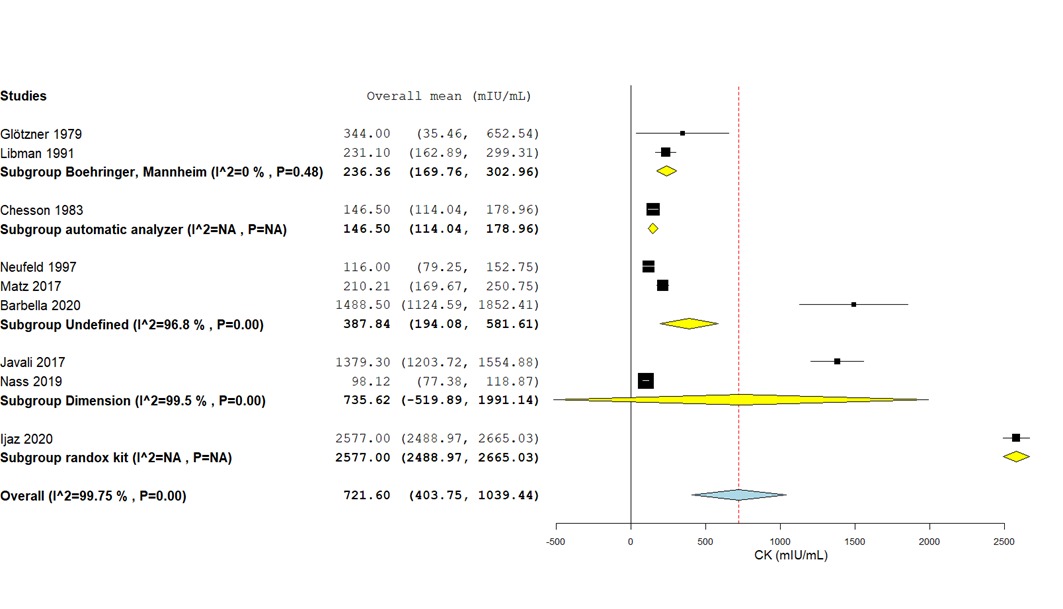** | **(c)** |

**Supplemental Figure 7. Subgroup analysis by mean age (a), country (b), and device of CK measurement (c) for mean CK level in GTCS patients at the first 0-6 hrs after seizure**

| **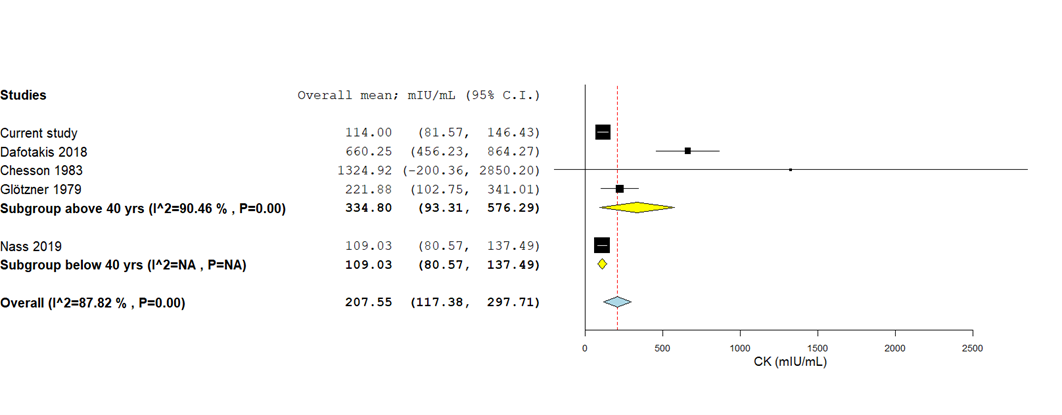** | **(a)** |
| --- | --- |
| **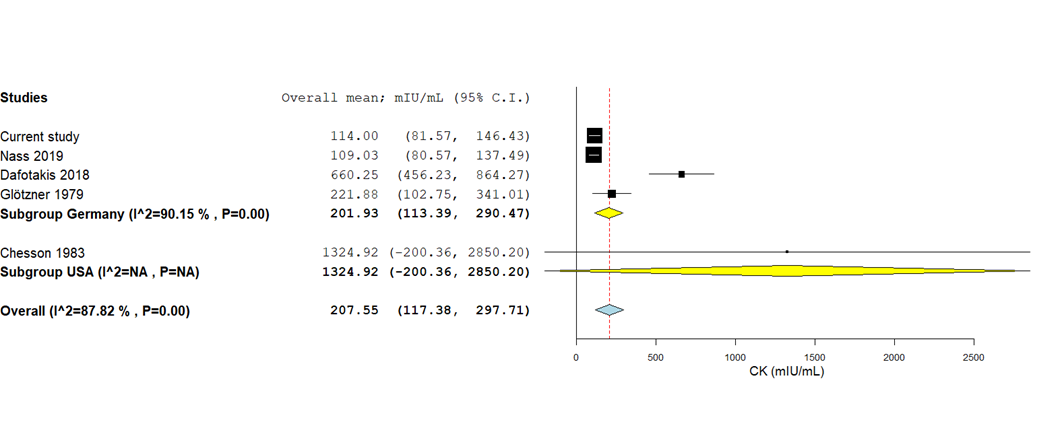** | **(b)** |

**Supplemental Figure 8. Subgroup analysis by age (a), and country (b) for mean CK level in GTCS patients at day 1 post-ictally**

| **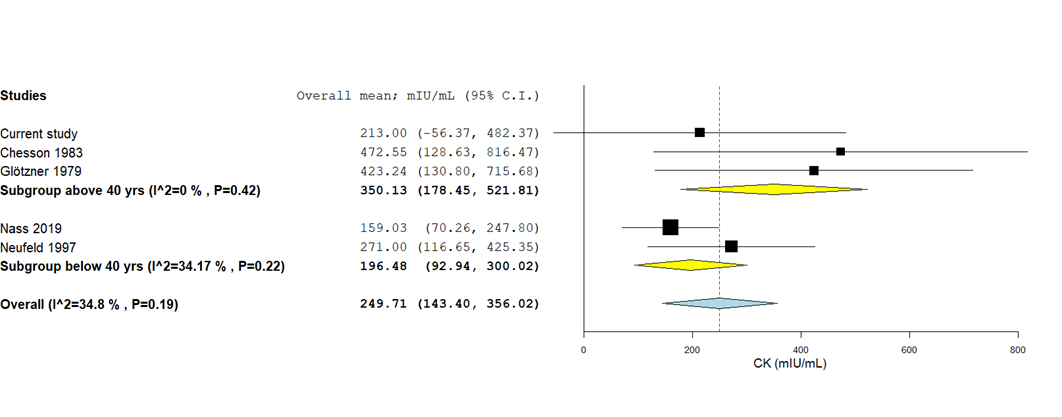** | **(a)** |
| --- | --- |
| **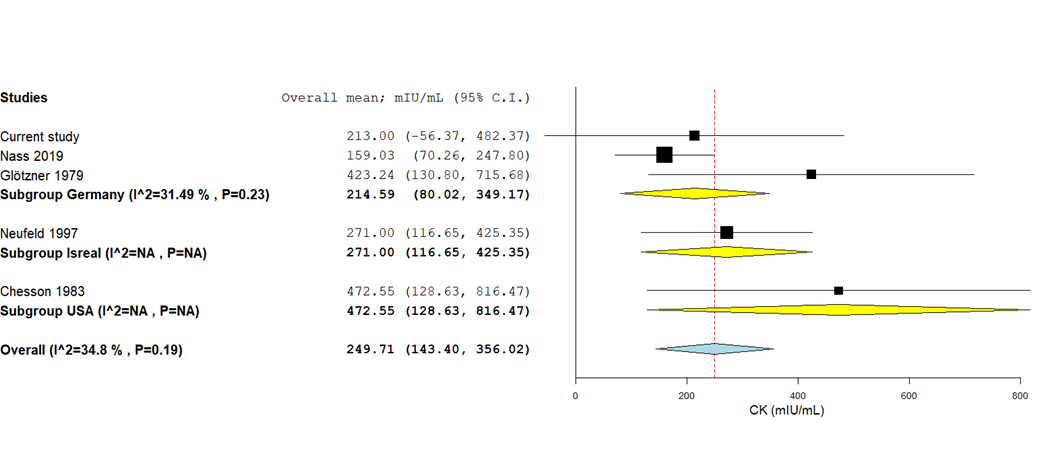** | **(b)** |

**Supplemental Figure 9. Subgroup analysis by mean age (a) and country (b) for mean CK level in GTCS patients at day 2 post-ictally**

**
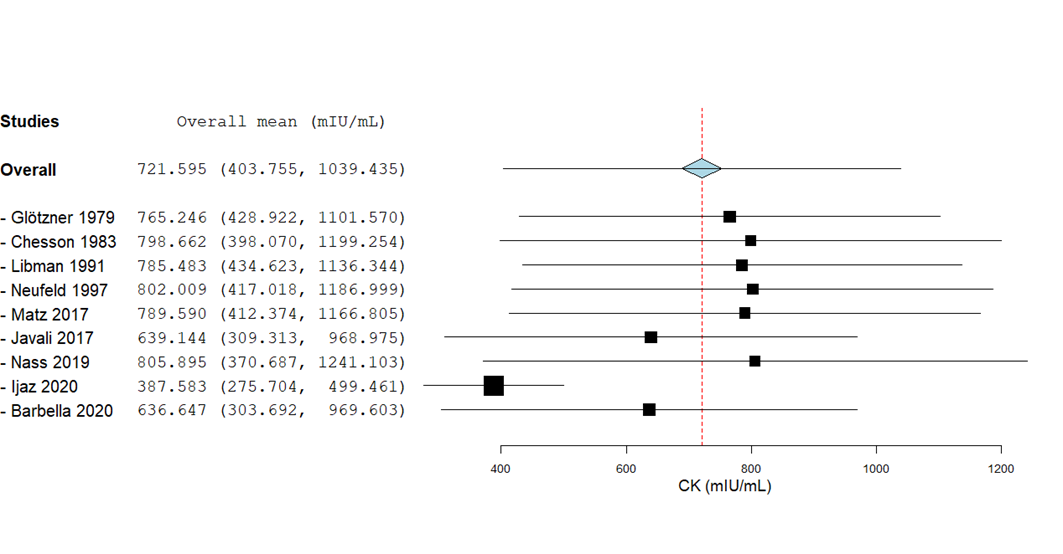
**

**Supplemental Figure 10. leave-one-out analysis for mean CK level in GTCS patients during the first 6 hours following the seizure**
